# Anthropogenic Barriers Limit Fish Access to Essential Habitats in the Amazon in the Face of Climate Change

**DOI:** 10.1111/gcb.70685

**Published:** 2026-01-02

**Authors:** Kátia Yasuko Yofukuji, Thomaz Mansini Carrenho Fabrin, Bruno Henrique Mioto Stabile, Angelo Antonio Agostinho, Céline Jézéquel, Valéria Flávia Batista‐Silva, Luiz Fernando Esser, José Hilário Delconte Ferreira, Reginaldo Ré, Pablo A. Tedesco, João Carlos Azevedo, Dayani Bailly

**Affiliations:** ^1^ Programa de Pós‐graduação Em Ecologia de Ambientes Aquáticos Continentais (PEA) Universidade Estadual de Maringá (UEM) Maringá PR Brazil; ^2^ Núcleo de Pesquisa Em Limnologia, Ictiologia e Aquicultura (Nupélia) Universidade Estadual de Maringá (UEM) Maringá PR Brazil; ^3^ Centre de Recherche Sur la biodiversité et L'environnement UMR 5300, Université de Toulouse, CNRS, IRD Toulouse France; ^4^ Programa de Pós‐graduação Em Biodiversidade e Sustentabilidade Ambiental (PGBSA), Grupo de Estudo Em Ciências Ambientais e Educação (GEAMBE) Universidade Estadual de Mato Grosso Do Sul (UEMS) Mundo Novo MS Brazil; ^5^ Universidade Tecnológica Federal Do Paraná (UTFPR) Campo Mourão PR Brazil; ^6^ CIMO, LA SusTEC, Instituto Politécnico de Bragança Bragança Portugal

**Keywords:** barrier prioritization, dams, fragmentation, freshwater fish, range shift, species distribution modeling

## Abstract

Barriers represent one of the greatest threats to river integrity and freshwater fish, as they fragment habitats and impair species dispersal, particularly in a scenario of climate change. In this context, we applied a novel framework that combined predictions of species distribution models with a river connectivity index to identify accessible and climatic‐environmental suitable habitats for frugivorous and socioeconomically important fish in the Amazon basin. We also ranked dams based on their potential for river fragmentation and blocking access to climate refuge for fish species that provide essential ecosystem functions and services in the Amazon. Our results revealed that there are still extensive areas that remain both connected and climatic‐environmentally suitable along the Amazon‐Solimões rivers, acting as core areas for fish dispersal and tracking suitable habitats. However, the planned expansion of hydropower infrastructure combined with climate change can lead to a contraction of areas that will remain simultaneously climatic‐environmental suitable and connected. By identifying and ranking the most impactful barriers, our results can provide innovative and applicable information for sustainable energy planning decisions in the Amazon. These results can inform policies and conservation actions aimed at preserving river connectivity, biodiversity, and ecosystem services under rapidly changing conditions.

## Graphical abstract

Rivers across the Amazon Basin are increasingly threatened by climate change and dam construction, which restrict fish movements. We combined climate projections with river‐network connectivity analyses to identify where suitable habitats for frugivorous and socioeconomically important fish are likely to persist and where access may be lost due to fragmentation. Our results show that climate change and dams act together to reduce connectivity, limiting access to future refuges. By identifying priority rivers and the most fragmenting barriers, our study informs conservation planning and sustainable energy development in the Amazon.

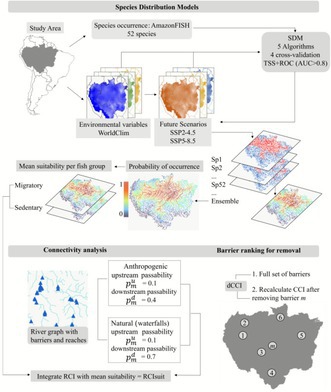

## 1 Introduction

For freshwater fish to track suitable habitats as anthropogenic barriers continue to expand across river basins might become an insurmountable challenge in the future (Radinger et al. 2018; Peluso et al. 2022; Franklin et al. 2024). The wide portfolio of existing barriers, such as dams, dikes, and weirs, is expected to expand further in the near future to meet demands of demographic and technological growth (Zarfl et al. 2015; Grill et al. 2019). Barriers can significantly disrupt river connectivity, obstruct fish movement, and alter habitat quality through changes in both physical and limnological characteristics while restricting access to spawning and feeding grounds (Agostinho et al. 2008; Seliger and Zeiringer 2018). Ultimately, river reaches fragmented by barriers will become spatially and functionally isolated for fish species, impairing recolonization processes and increasing the risk of local extirpations (Carvajal‐Quintero et al. 2017).

The location and attributes of barriers (e.g., size, passability), especially dams, determine the extent and severity of their impacts (Pelicice and Agostinho 2008; Rodeles et al. 2020). Dams that prevent access to floodplains or river reaches highly connected to large tributaries have particularly severe ecological consequences. Fish are often unable to move upstream past barriers, even when fishways exist (Lira et al. 2017; Zarri et al. 2022). The effectiveness of these structures installed to facilitate fish movement, including dispersal and migration, is questionable and varies considerably across species and site conditions (Pelicice and Agostinho 2008; Silva et al. 2017). Even when fish by‐pass dams, the large reservoirs upstream are unsuitable for most rheophilic fish, trapping them in habitats that reduce their fitness (Pelicice and Agostinho 2008). Downstream movements can also be challenging as eggs can settle to deeper parts of the reservoir, juveniles can be easily predated, and there is also a high risk of mortality for fish passing through hydroelectric turbines (Agostinho et al. 2021). Addressing the impacts of installed and planned hydroelectric plants with respect to their position in the river network is therefore fundamental to understand hydrological fragmentation, providing relevant information for environmental planning and management. Despite the plethora of implications of river fragmentation, conservation and restoration of aquatic systems often overlook connectivity measures (Rodeles et al. 2020; Thieme et al. 2023).

Megadiverse basins such as the Amazon are currently threatened by the escalating effects of climate change and river fragmentation (Hurd et al. 2016; Herrera‐R et al. 2020). The Amazon basin has a strong impact on global climate because of its historically high rates of evapotranspiration, precipitation, and river discharge (Marengo 2005; Marengo et al. 2024). Hydrological instability caused by climate change, coupled with increased damming, may trigger severe alterations in the Amazon water cycle. As plans for hydropower expansion intensify in tropical regions, the Amazon basin is expected to have the highest number of newly built dams in the near future (Zarfl et al. 2015; Flecker et al. 2022). Amazonian fish species, many of which are of commercial interest, depend on river connectivity for completion of their life cycles (Röpke et al. 2015; Arantes et al. 2017) and for moving to future climate refugia (Radinger et al. 2018). Consequently, many human communities and their livelihoods rely on maintaining river connectivity as a precondition for the regular supply of ecosystem services, including fish harvests.

Among fish, species with fruit‐eating habits stand out as vital from ecological and socioeconomic points of view as they are major inland fisheries targets and play unique roles in the supply of an array of ecosystem services (Anderson et al. 2009; Nagl et al. 2021). These species provide food security in most local traditional and indigenous communities which have a fish‐based diet (Isaac et al. 2015; Begossi et al. 2019). In addition, frugivorous species, such as *tambaqui* ( *Colossoma macropomum* ) and *matrinxã* ( *Brycon amazonicus* ), are of high commercial importance owing to their large body size and high appreciation by human consumers, making them relevant and valuable sources of income for Amazonian people (Anderson et al. 2011; Hallwass and A.M. Silvano 2016). From an ecological functional perspective, frugivorous fish are essential in the maintenance of forest ecosystems through seed dispersal (Correa et al. 2016). Fish can move long distances, facilitating the colonization of remote patches and connecting plant populations in fragmented landscapes, maintaining regional forest diversity (Anderson et al. 2011; Nagl et al. 2021).

Protecting and restoring river connectivity will be the cornerstone of climate adaptation in a world facing high rates of habitat fragmentation (Thieme et al. 2023; Stoffers et al. 2024). However, conservation efforts to maintain free‐flowing rivers will not successfully protect freshwater fish biodiversity unless they integrate species' future distributions with the portfolio of projected barriers that will affect dispersal to suitable environments in the future. Similarly, restoring free‐flowing rivers by removing or retrofitting barriers may not be as effective as expected if exclusively focused on structural connectivity (i.e., based on the physical structure of the river network), neglecting the redistribution of species in response to climate change (Kemp and O'hanley 2010). For that reason, enhancing dispersal to high‐quality habitats based on species requirements for future climate scenarios might be much more effective than reconnecting long stretches of low habitat suitability (Rodeles et al. 2020; Stoffers et al. 2024; Wegscheider et al. 2024). Integrating climate change and river fragmentation within the same framework is thus urgent to address synergistic effects and to support more effective river conservation and restoration efforts. If climatically suitable habitats for fish are unreachable due to barriers, ecosystem processes, function, and services will be disrupted, increasing the vulnerability of traditional human communities that depend on them.

A previous study addressed river connectivity in the Amazon basin focused on identifying connectivity corridors for freshwater species, including some long‐distance migratory fish (Caldas et al. 2022). However, it did not predict the potential distribution ranges of the species for the present and the future. Flecker et al. (2022) focused on identifying portfolios of sites which minimize the impact of dams on river connectivity and fish diversity under current climate. Although some studies have addressed climate change, they adopted a more regional approach focused on the Andean Amazon (Anderson et al. 2018; Herrera‐R et al. 2020). The goal of our study was to evaluate the interplay of climate change and barrier‐driven river fragmentation on frugivorous fisheries resources across the Amazon basin focused on the accessibility of suitable areas in the years to come. The research questions were: (1) how frugivorous fish species will redistribute in the future in response to changes in climate? (2) how does present and future barrier establishment constrain or enable access to climatically suitable habitats? and (3) which existing and planned barriers contribute most to reducing access to climatically suitable areas? By identifying the most impactful installed and planned hydroelectric plants in the Amazon, this study contributes to informing the sustainable planning of the energy matrix expansion in order to minimize impacts on natural capital in the face of climate change. Ultimately, this study will contribute to decision‐making in processes related to the continuous provision of valuable ecological, social, economic, and cultural benefits provided by Amazonian frugivorous fish.

## 2 Material and Methods

**FIGURE 1:**
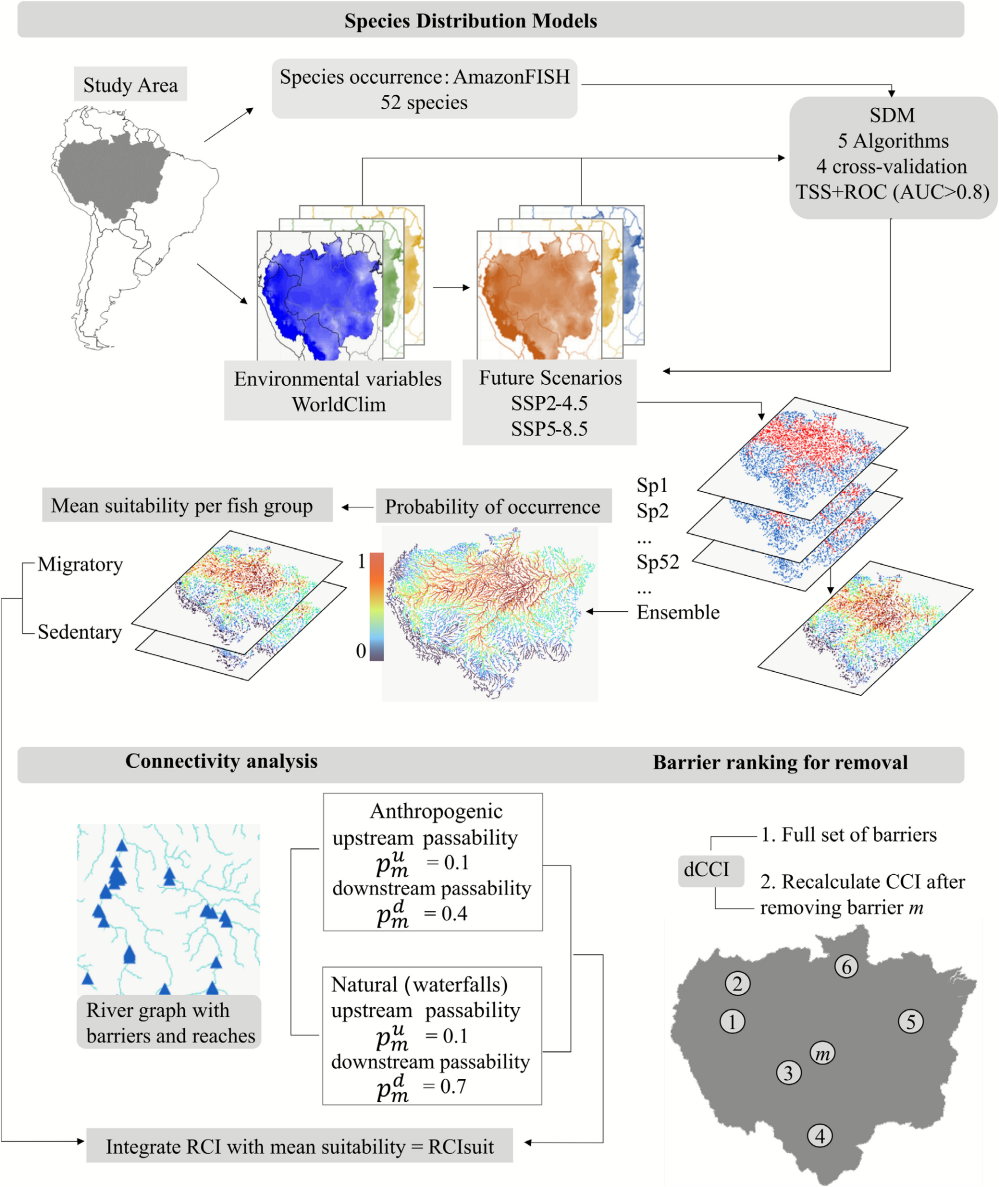
Schematic representation of the modeling workflow consisting of Species Distribution Models (top), Connectivity analysis (bottom left), and Barrier prioritization (bottom right) for current and future barriers, considering climatic‐environmental suitable habitats of 52 fish species of the Amazon basin. CCI, Catchment Connectivity Index; RCI, Reach Connectivity Index; RCIsuit, Reach Connectivity Index weighted by suitability; SSP, Shared Socioeconomic Pathway; *dCCI*, gain in connectivity by barrier removal.

We followed a series of modeling steps to prioritize climatically suitable and connected habitats for fish communities at the Amazon basin scale (Figure 1; Supporting Information—ODMAP Protocol). First, we modeled the potential distribution of 52 frugivorous fish species of socioeconomic value (Table S1) for both the present (1970–2000) and the future (2030–2100), under two climatic scenarios (moderate: SSP2‐4.5 and pessimistic: SSP5‐8.5). Next, using a graph‐based approach, we modeled the connectivity of the Amazon basin, including the effect of current barriers (waterfalls and existing anthropogenic barriers) and future projected infrastructures (a combination of current and proposed barriers). Finally, we assessed the contribution of individual barriers that most significantly fragment the Amazon basin.

### 2.1 Species Selection and Grouping

Fish were selected based on frugivory level and with respect to their value to fisheries, resulting in 52 species (for further details see Supporting Information). We arranged these species into two groups based on their movement behavior. The migratory group included 27 species (Table S1) that demonstrate some level of migration. Such species rely on basin‐wide connectivity between rivers and distinct habitats to access feeding areas and complete life‐cycle events such as reproduction and development (Duponchelle et al. 2016; Herrera‐R et al. 2023). The sedentary group included 25 species (Table S1) that are primarily residents and some that exhibit restricted lateral migration into floodplains or flooded forests during high‐water periods (Arantes et al. 2021). Finally, we considered the combined group of both migratory and sedentary species to examine a general response from the 52 species.

### 2.2 Spatial Framework

**FIGURE 2:**
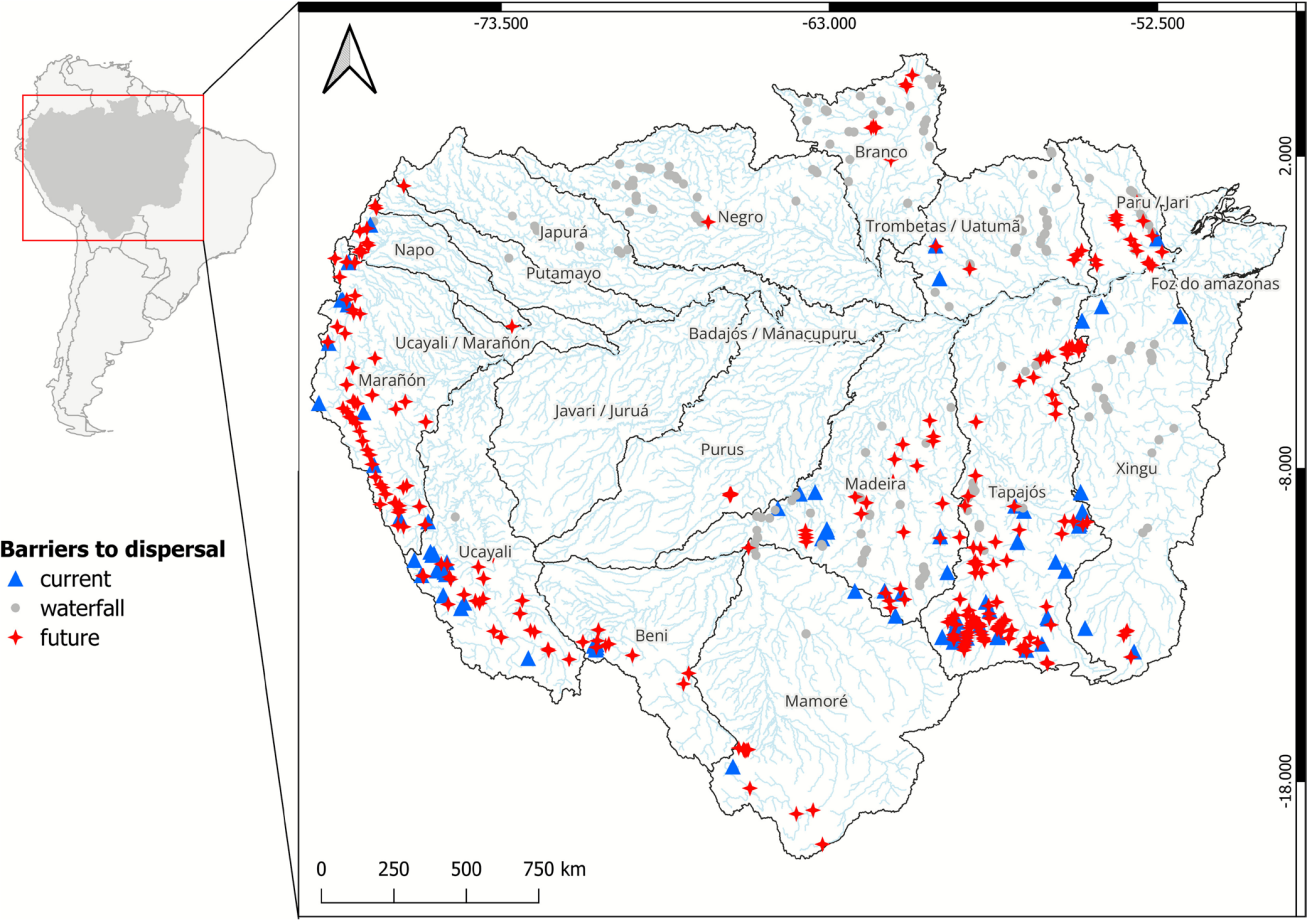
Amazon basin with the location of anthropogenic and natural barriers to fish dispersal. Barriers data were collected from multiple sources, as detailed in Table S2. Map lines delineate study areas and do not necessarily depict accepted national boundaries.

Our research area encompasses the entire Amazon basin, including regions from Bolivia, Brazil, Colombia, Ecuador, Guyana, and Peru (Figure 2). The Amazon, covering over 6 million km^2^, is the largest river basin in the world and contributes to nearly 20% of the planet's freshwater discharge. It harbors four of the world's 10 largest rivers: the Amazon mainstream and its tributaries Negro, Madeira, and Japurá. For our analysis, basin and river network spatial data were obtained from the HydroRIVERS database (Lehner and Grill 2013). The barriers in the analysis of fragmentation were comprised of natural barriers, that is, waterfalls, and anthropogenic barriers (e.g., dams, weirs, culverts) (Table S2; Figure 2). All present‐day barriers were validated by means of the inspection of high spatial resolution remote sensing images to confirm their existence and to ensure they effectively block the river. Future barriers were cross‐checked against official government documents, including energy expansion plans and environmental study inventories, to validate their inclusion in future scenarios.

### 2.3 Species Distribution Modeling

Climatic‐environmental suitability for the occurrence of frugivorous species in the Amazon was obtained from the species distribution modeling technique (for detailed modeling workflow, please see the Supporting Information—ODMAP protocol). This approach associates, in a correlative sense, the occurrence records of the species with variables representing the environmental space of a given study area to estimate climatic‐environmental conditions that are suitable for the species occurrence across the geographic space. For each considered species, the climatic‐environmental suitability was obtained for the current and future times, projecting the outputs on the hydrographic network of the Amazon River basin encompassing rivers of order 4 or larger, based on the information provided by the HydroSHEDS database (https://www.hydrosheds.org/products/hydrorivers). The distribution of the species was modeled as a function of bioclimatic and hydrological variables (Table S4) covering the Amazon hydrographic network. Present and future (2030, 2050, 2070, and 2090) bioclimatic variables were extracted from the WorldClim 2.1 database (http://www.worldclim.org/) based on two Shared Socio‐economic Pathways (SSPs), one moderate (SSP2‐4.5) and one pessimistic (SSP5‐8.5). We used five algorithms to predict the environmental suitability and potential distribution of species in the study area. From the outputs of such algorithms, and considering the criterion adopted for evaluating the predictive performance of models, we obtained the consensus model (see details on Supporting Information—ODMAP protocol). Predictive performance of ENMs was evaluated using the Area Under the Receiver Operating Characteristic Curve (AUC), retaining those with AUC > 0.8, which were combined to obtain a consensus prediction (Araújo and New 2007). The presence‐absence outputs of the consensus model, representing the potential distribution of the species, were obtained following the majority consensus rule. This means that models were binarized with the threshold that maximizes the sum of sensitivity and specificity, and then cells in which at least more than half of the models indicate the presence of the species were considered occupied. All maps were constructed on a regular geographical grid with 40,222 cells with a spatial resolution of 7 km covering the entire river network.

### 2.4 Connectivity Indices Assessment

To evaluate longitudinal connectivity within the Amazon basin for fish at present and for future time, we followed recommendations outlined in Baldan et al. (2022). Using hydrographic data, the river network was represented as a graph, where edges represent barriers or confluences, while nodes correspond to river reaches (Baldan et al. 2022). A “river reach” was defined as a unit represented by a segment between two neighboring confluences or barriers. Connectivity was quantified as the probability of dispersal between two reaches within the river network. For the connectivity analyses, we employed the *riverconn* R package (Baldan et al. 2022), which provides connectivity indices integrating the number, passability, and position of barriers. We calculated the Reach Connectivity Index (RCI) as the weighted sum of the probabilities of dispersal (*I*_*ij*_) between a specific reach *i* and every other reach *j* in the river network:

(1) $$\mathrm{RC}I_{i}=\sum_{j=1}^{n}I_{\mathrm{ij}}\frac{w_{j}}{W}$$

where *n* is the number of reaches in the network, *w*_*j*_ is the weight of the reach *j*, and *W* is the sum of the weights over the *n* reaches. In our study, we used the river length as reach weights. This index can describe structural connectivity only if accounting for the number and spatial arrangement of barriers within the network ($c_{\mathrm{ij}}$) as follows:

(2) $$I=c_{\mathrm{ij}},$$

where

(3) $$c_{\mathrm{ij}}=\prod_{m=1}^{k}p_{m}^{\mathrm{eq}},$$

where $p_{m}^{\mathrm{eq}}$ is the estimated passability of a barrier *m*, depending on the direction (upstream or downstream) in which it is encountered, combined with all *k* barriers in the path. Asymmetric directionality was set to “outgoing” mode to represent the potential for fish to disperse from a given reach to other regions in the river network.

To calculate RCI values, a numerical value for the passability, either up‐ or downstream, of each barrier is needed. Passability can range from 0 to 1, where 0 represents an impassable barrier while 1 indicates full passability. We estimated up‐ and downstream passability values based on expert judgment regarding the overall capability of the Amazonian fish species to overcome barriers and assuming a conservative estimate based on the low efficiencies of fish passage for dams and species from South American rivers (Makrakis et al. 2007; Pelicice and Agostinho 2008; Hahn et al. 2020). Therefore, we set up‐ and downstream passabilities of anthropogenic barriers to 0.1 and 0.4, respectively. We set a low downstream passability for anthropogenic barriers, as downstream dispersal can also be disrupted. Fish that disperse upstream from a barrier hardly return, except for those that return through turbines and spillways, but the mortality rate is usually high. Eggs and larvae can drift downstream barriers, but particularly for reservoirs, water residence time can affect survival rates for this life stage (eggs can sediment, and mortality is high) (Pelicice et al. 2015). For natural barriers (waterfalls), we set the upstream passability to 0.1 and the downstream dispersal to 0.7.

In order to evaluate robustness to uncertainty in expert‐elicited passability values, we recomputed RCI for all river reaches and fish groups under alternative combinations of upstream‐downstream passability by 10%–25% around baseline values. Climatic‐environmental suitability was kept fixed at current time so that only the passability parameter varied. For each scenario we compared RCI values with the reference using Pearson correlation and paired Wilcoxon signed‐rank tests. These showed that RCI values remained congruent across moderate perturbations (*r* > 0.97). Only the restrictive scenario, with fully blocked upstream passability, showed significant differences (Table S3).

Then, to integrate structural connectivity with climatic‐environmental suitability we multiplied the RCI values by the mean suitability predicted for each fish group. For that, we calculated the mean suitability across all modeled species within each group (combined, migratory, and sedentary). This generated a group‐level suitability layer in which each grid cell represents the average climatic‐environmental suitability of the species group. This integration provides a composite measure that reflects both the accessibility of habitats (RCI) and their climatic‐environmental suitability, ensuring that regions favorable for fish dispersal were identified based on both criteria. This integrated index (RCIsuit) ranges from 0 to 1, where higher values indicate higher levels of longitudinal connectivity and suitable areas, and lower values represent lower connectivity of river reaches and climatic and environmentally unsuitable areas.

To assess the relative importance of barriers (waterfalls and anthropogenic) for basin‐level connectivity, we used a barrier prioritization simulation, which prioritizes infrastructures whose removal (or prevention of construction, for future barriers) improves connectivity at the Amazon basin scale (Baldan et al. 2022). For this, we used the “leave‐one‐out” approach, where the value of a Catchment Connectivity Index (CCI) is calculated first based on the full setup with all barriers (CCI_start_) then recalculated after removing barrier *m* (CCI_start,m_), and finally the difference between the two values expressed as a proportion of the CCI before barrier *m* removal:

(4) $$\mathrm{dCC}I_{m}=\frac{\mathrm{CC}I_{\text{start},m}-\mathrm{CC}I_{\text{start}}}{\mathrm{CC}I_{\text{start}}}100$$

CCI is calculated as the weighted sum of the RCI index at the catchment level, using mean suitability (current and future) as the weighting factor. dCCI ranges from 0, when the barrier *m* has no effect over landscape fragmentation, and infinite, when the catchment is totally fragmented. Values of dCCI can therefore be ranked and used to prioritize barriers based on their contribution to overall fragmentation.

Finally, to gain insight on the effects of climate change and the future expansion of anthropogenic barriers proposed for the basin, we ran the simulations using two distinct setups: the “current barrier,” where only existing barriers and waterfalls were included (i.e., infrastructure constructed up to 2024) and changes on connectivity were analyzed by altering habitat suitability values across different years and SSPs (moderate and pessimistic); and the “future barrier” setup, where we added the proposed future barriers to the current set along with the existing waterfalls, and assessed the combined impact of these new barriers and changing habitat suitability over time. These simulations contribute to a comprehensive assessment of future conditions, highlighting the potential connectivity challenges under the pressure of shifting bioclimatic conditions.

## 3 Results

### 3.1 Spatial Patterns From Species Distribution Modeling

Currently, the mean climatic‐environmental suitability for the occurrence of 52 species (combined group) is highest along the Amazonas‐Solimões mainstem, the middle and lower Madeira and Tapajós, but also on Purus, Trombetas/Uatumã, Branco and Negro subbasins (Figure S1). For some migratory species, some tributaries of Beni, Mamoré, Ucayali, and Marañón were also predicted to be highly suitable for the current time (Figure S1). Towards the end of the century, projections indicate that the central region of the basin will continue to harbor the majority of highly suitable areas. However, substantial contraction of highly suitable areas from the central region is projected, especially under the pessimistic scenario, with high suitable areas becoming increasingly restricted to some tributaries of the lower Madeira, Tapajós, and Negro subbasins (Figures S2–S4).

### 3.2 Effects of Barriers and Climate Change on River Connectivity

**FIGURE 3:**
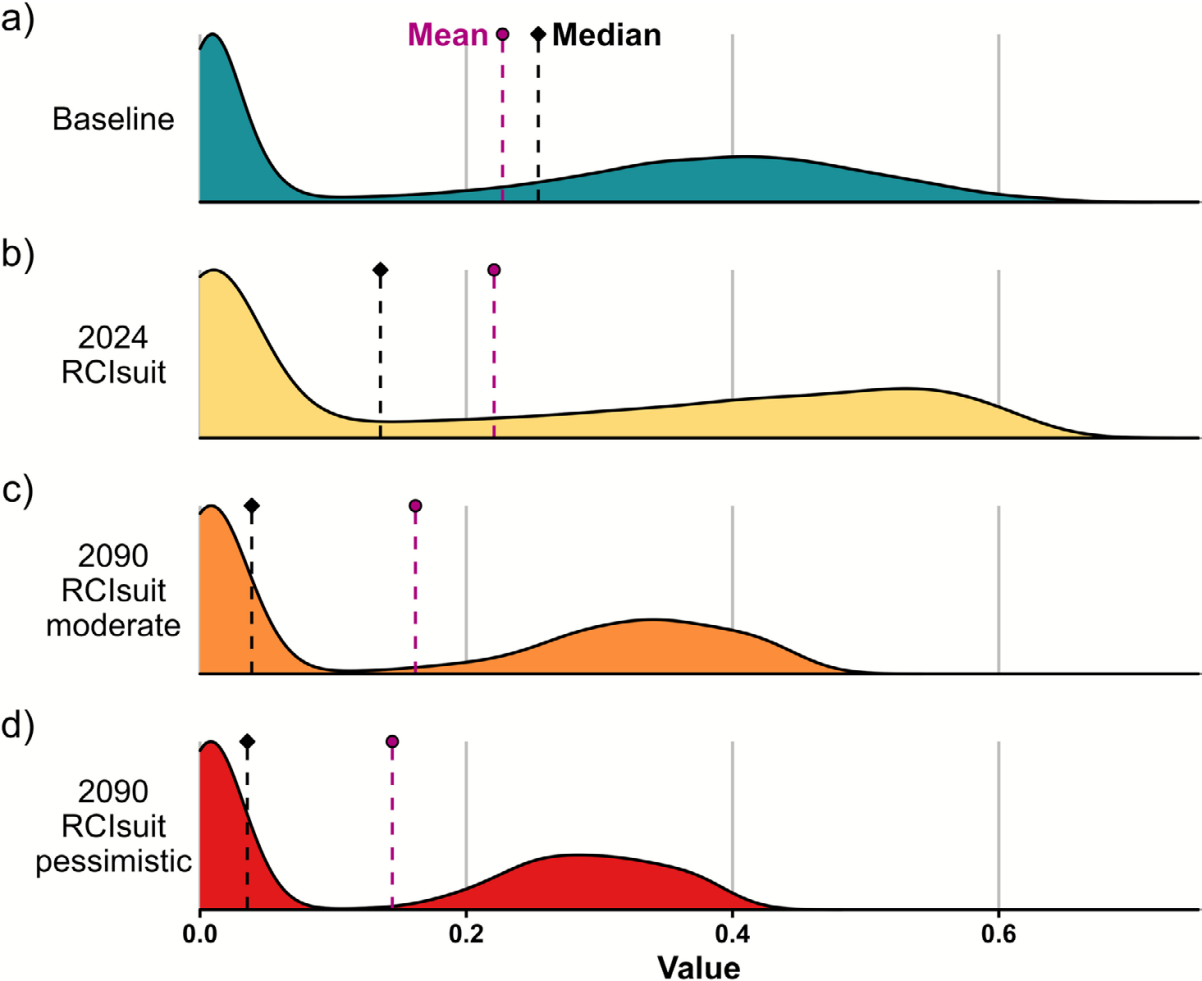
Distribution of RCIsuit values for the combined fish group under four simulations: Baseline (represents natural fragmentation only by waterfalls combined with current climatic‐environmental suitability), 2024 RCIsuit (corresponds to the combination of existing dams and waterfalls under current climatic‐environmental suitability), and 2090—moderate and pessimistic (represents the addition of proposed barriers to the current set under changing climatic‐environmental suitability of the end of the century). RCIsuit values integrate the accessibility of habitats and their climatic‐environmental suitability, ensuring that regions favorable for fish dispersal were identified based on both criteria. Each density plot represents the distribution of values with summary statistics: Dashed black line marks the median, and the dashed purple line indicates the mean value for each distribution.

Our analysis revealed varying degrees of habitat fragmentation across barriers (current and future) and climatic (moderate and pessimistic) scenarios with a marked asymmetry in the distribution of RCIsuit values (Figure 3; Table S6). Under baseline conditions (i.e., natural fragmentation only by waterfalls and current climate), RCIsuit values tend to be higher, as indicated by higher maximum and mean values (mean = 0.23 and max 0.66 for the combined fish group, mean = 0.22 and max = 0.65 for the sedentary and mean = 0.23 and max = 0.67 for migratory fish groups; Table S6). Under the current barrier setup, we observed an increase in the median of RCIsuit over the century, while the mean and maximum RCIsuit decreased (Table S6). This results from an increase over time in the frequency of RCIsuit values due to shifts in species potential distribution. However, values close to the maximum (i.e., those indicating environmentally suitable and connected areas) become less frequent, indicating an overall loss of connectivity in the most favorable areas.

This loss becomes more pronounced under the future barrier setup, especially under the pessimistic scenario, which incorporates both projected barrier construction and climate‐induced shifts in habitat suitability (Table S6; Figures S5 and S6). For example, for the combined fish group, median RCIsuit decreased from 0.13 (2024) to 0.039 (2090) under the moderate scenario and to 0.035 in the pessimistic scenario. Mean RCIsuit also decreased, ranging from 0.22 (2024) to 0.16 (2090) in the moderate scenario and from 0.22 (2024) to 0.14 (2090) in the pessimistic scenario (Table S6). Notably, in the pessimistic scenario, the maximum RCIsuit value, which initially reached nearly 0.51 in some areas, decreases to 0.41 by 2090. Additionally, these higher values become even less frequent, as indicated by the decreasing density of maximum values in the density plots (Figure 3a–d).

**FIGURE 4:**
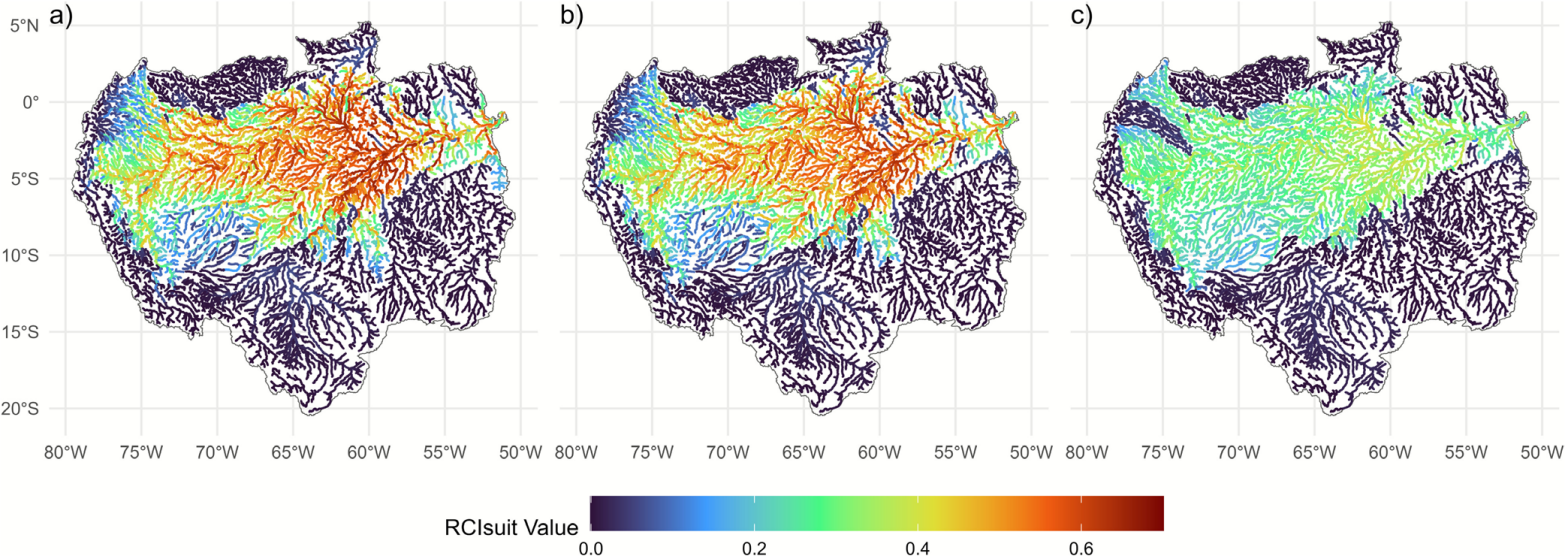
Example of spatial patterns of RCIsuit across the Amazon basin for the (a) baseline (current climate and natural fragmentation by waterfalls), (b) current, and (c) future barrier sets under the pessimistic climate scenario for 2090, shown for the combined fish group. The color scale for RCIsuit ranges from red (high connectivity and suitable habitats) to dark blue (low connectivity and unsuitable habitats). Map lines delineate study areas and do not necessarily depict accepted national boundaries.

Our results show that the Central Amazon region currently exhibits relatively high RCIsuit rankings, which indicates that this area hosts environmentally suitable river habitats that remain highly connected under baseline conditions (Figure 4a). When present‐day barriers are considered (Figure 4b), connectivity decreases noticeably in parts of the Madeira and Xingu basins (Figure 4d), directly reflecting the fragmentation imposed by existing dams. Rivers located in the far west (Ecuador and Peru), such as in Ucayali and Marañon, are fragmented, as shown by RCIsuit values close to zero, and are currently climatic and environmentally unsuitable across all fish groups (Figure S1; Figure 4b). Further south, the mid and upper reaches of Madeira, Tapajós, and Xingu subbasins in Brazil are also essentially disconnected from the central Amazon, as shown by low RCIsuit (Figure 4b). This fragmentation highlights critical disconnections in areas that otherwise present climatic‐environmental suitable habitats, especially for migratory fish (Figure S2). For sedentary species, present‐day connectivity is more favorable in the central‐eastern Amazon, which exhibits higher RCIsuit values and is predicted to have better climatic‐environmental suitable conditions (Figure S3). Building all proposed barriers combined with projected changes of suitable habitats under future climate scenarios reveals further declines in connectivity, with alarming trends observed in the Napo subbasin, as indicated by RCIsuit close to zero (Figure 4c; Figure S5). Overall, under the pessimistic climate scenario, suitability for all fish groups tends to decline over the century (Figures S2–S4), amplifying the general downward trend in RCIsuit, with climatic‐environmental favorable areas increasingly being cut off by planned barriers (Figure 4b–e; Figure S6).

### 3.3 Barrier Prioritization for Increasing River Connectivity

Currently, nearly 300 dispersal barriers have been validated across the Amazon basin, including both natural waterfalls and anthropogenic infrastructures. Some subbasins concentrate most of the existing barriers, such as the Madeira (12), Tapajós (31), Ucayali (14), and Marañón (10). For the future, the number of anthropogenic barriers will be up to three times higher than it is today, with an additional 254 barriers proposed or in different planning stages, especially in the western and southeast of the basin. Tapajós, Marañón, Ucayali, and Madeira will be the most affected subbasins with, respectively, 129, 55, 38, and 35 new dams.

**FIGURE 5:**
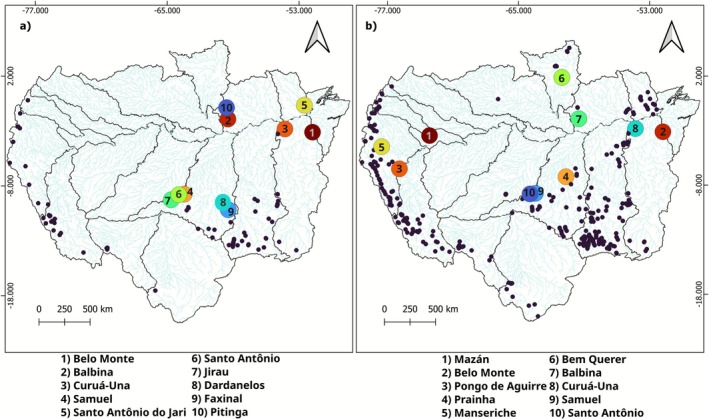
Mean ranking of reservoir impact considering their potential for hydrological fragmentation and blocking access to climate refuge areas for fish species in Amazon for (a) current and (b) future barriers across climate scenarios (moderate and pessimistic) and fish groups (migratory, sedentary, and combined). The top 10 most impactful barriers are highlighted in the figure, indicating the highest‐ranked barriers which decrease overall basin connectivity. Rank 1 corresponds to the most impactful hydroelectric plant, rank 2 corresponds to the second hydroelectric plant with the greatest impact, and so on. Map lines delineate study areas and do not necessarily depict accepted national boundaries.

Barriers with the greatest impact on river connectivity are at key nodes of the river network, particularly where they block access to extensive upstream habitats (the rank of impact provided by the current and future hydroelectric plants is available in the Data S1). Dams located on large tributaries, such as Belo Monte in the Xingu, Curuá‐Una in Curuá‐Una, and Balbina and Pitinga in the Uatumã, disrupt dispersal from nearly the entire subbasins (Figure 5a). In the Madeira basin, which concentrates half of the 10 most impactful barriers in the Amazon region, Jirau and Santo Antônio are situated along the mainstem, while Samuel, on the Jamari River, a tributary, further fragments connectivity by isolating a highly connected upstream network (Figure 5a). Future scenario analyses indicate that some of the existing dams will continue to be among the 10 most impactful barriers (e.g., Belo Monte, Balbina, Curuá Una, Samuel, Santo Antônio). Considering the climatic‐environmental suitability shifts, some barriers will change their relative rank position through time (Data S1). The proposed Mazán dam, the only planned project of Napo in Peru, poses a risk of disconnecting the entire watershed by damming the mainstem above its confluence with the Amazon River, becoming the top 1 most impactful barrier in all scenarios and fish groups (Figure 5b). Belo Monte also consistently appears as the second most impactful dam. Pongo de Aguirre, initially ranked fourth, rises to third position by the end of the century, while Prainha shifts from third to fourth. The Tambo 60, particularly under the pessimistic scenario, rises in the ranking, becoming increasingly fragmenting and entering the top 10. In the Branco subbasin, the planned Bem Querer dam (ranked sixth) threatens to disconnect large northern reaches that are suitable for fish (Figure 5b). Barriers in the highest positions in the rank tend to remain relatively stable across scenarios, whereas those in lower positions show greater viability, reflecting fluctuations in climatic suitability and changes in their influence on basin connectivity (Data S1).

Fragmentation is most severe in the Brazilian lowlands, particularly in the Madeira and Tapajós, where habitat is currently climatic‐environmental suitable for fish. Proposed dams like Prainha (4th), Cachoeira Galinha, Tabajara, and Sumaúma in the Madeira subbasin are expected to fragment vast areas of climatically suitable habitat. Tapajós, already highly fragmented, is the subbasin where the largest number of new dams is planned, posing additional risks to connectivity across the already affected mainstem and major tributaries.

## 4 Discussion

Our results offer a basin‐scale perspective of important undammed reaches supporting current and future suitable conditions for the occurrence of 52 fish species ecologically and socioeconomically important in the Amazon. They align with broader evidence that the disordered hydropower expansion threatens basin‐wide connectivity, placing both fish populations and the human communities that depend on them in a grievous vulnerability condition. Despite the expansion of hydropower infrastructure and expected changes in habitat suitability driven by climate change, there are still extensive areas that remain both hydrologically connected and climatic‐environmentally suitable, particularly along the Amazon‐Solimões rivers. These regions act as core areas for fish dispersal and tracking suitable habitats, even under pessimistic climate change scenarios. Preserving these areas is therefore pivotal to maintain ecological processes and the provision of resources for traditional and indigenous communities.

Many subbasins in the Amazon already exhibit signs of hydrological disconnection. The Madeira, Tapajós, and Xingu subbasins, as well as tributaries in the west (e.g., Ucayali and Marañón subbasins), show very low RCIsuit values, indicating that even climatically suitable areas are no longer functional due to impaired connectivity. This is especially concerning for long‐distance migratory species, which migrate from the main channel and tributaries to complete their life cycles (Duponchelle et al. 2016; Herrera‐R et al. 2023). For resident fish or species with reduced dispersal capacity, barriers can often be impassable, and fish end up confined within short reaches, experiencing increased population densities, competition for resources, and a higher probability of local extirpation (Hurd et al. 2016; Zarri et al. 2022). Moreover, barriers often break the lateral connectivity between river channels and floodplains, which are seasonally flooded habitats essential for frugivorous fish during the high‐water season, when they enter the flooded forests or *igapós* to forage on fruits and seek refuge (Costa‐Pereira and Galetti 2015; Correa et al. 2016; Costa‐Pereira et al. 2017).

Our projections indicate that the planned expansion of hydropower infrastructure will markedly worsen this scenario. Under future climate scenarios, particularly the most pessimistic, we observed an expressive decline in maximum and mean RCIsuit values, indicating that areas that are simultaneously climatic‐environmental suitable and connected will contract. Therefore, additional barriers in middle and lower Madeira and Tapajós are strictly discouraged, as they not only fragment the mainstem and turn contiguous into isolated reaches, but also impact free‐flowing tributaries, which can cause losses of alternative routes for spawning and nurseries. These tributaries can ensure drift and development of eggs and juveniles, enabling fish recruitment across vast downstream areas (Casarim et al. 2018; Vasconcelos et al. 2020). Conversely, barriers placed farthest away to the west of Marañón and Ucayali might be less impactful when considering river connectivity alone. However, beyond river fragmentation, placing new barriers in these locations can potentially affect species by altering sediment and nutrient supplies from reaching downstream lowlands and their floodplains, causing drastic changes in sediment‐rich flows that sustain productivity in the basin (McClain and Naiman 2008; Finer and Jenkins 2012).

To safeguard freshwater biodiversity and sustain ecosystem services amid global changes, our study advocates halting the construction of new barriers or strategically removing those where environmental costs outweigh any potential benefits (Tickner et al. 2020; Thieme et al. 2023). For instance, Belo Monte and Balbina, respectively ranked as the first and second most impactful anthropogenic barriers currently installed in the Amazon, and also ranked among the most fragmenting barriers in the future, demonstrate the scale of socio‐economic and socio‐environmental impacts by suppressing river connectivity and processes dependent on periodic flooding. Although the decommissioning of such large dams is unlikely in the near term, enhancing basin‐wide connectivity may still be achievable through more feasible alternatives. By identifying and ranking smaller barriers that contribute the most to fragmentation, our analysis can support which existing barriers can be targeted for removal, and which planned infrastructure should be avoided, as smaller barriers often have disproportionately large cumulative impacts (Couto et al. 2021; Nickerson et al. 2022). However, such actions must be informed by comprehensive environmental assessments that account for sediment release, invasive species proliferation, and socioeconomic impacts (Bellmore et al. 2019; Habel et al. 2020).

Considering the future scenario of reservoir construction in the Amazon, our study reveals important threats to the access of fish to large and crucial suitable habitats. The construction of the Mazán dam on the Napo subbasin in Peru, ranked as the most impactful anthropogenic barrier in the future, will disconnect the entire watershed, affecting indigenous subsistence fisheries. Similarly, the Manseriche dam will further isolate fish populations between the Andes and the Amazon mainstem. In the Branco subbasin, north of Brazil, the planned Bem‐Querer dam will disconnect upper regions between the confluence with Rio Negro, which is composed of many conservation units, including sustainable use (Goulding and Barthem 2003). As for Madeira, the construction of proposed dams like Prainha, Cachoeira Galinha, Tabajara, and Sumaúma will confine fish populations between barriers, preventing fish from reaching upper stretches, with negative spillover effects on fisheries in Bolivia and Peru. These examples highlight how barriers on the mainstem or major tributaries can collapse ecological connectivity across entire subbasins, a situation that is even more worrying when climatic conditions in such areas remain suitable for fish persistence in the face of climate change. These results emphasize the relevance of our study in providing innovative and essential information for the sustainable planning of the expansion of the energy matrix in the Amazon. In this sense, we advocate that the effective construction of those highly impactful projects must be seriously weighed, balancing their economic benefits from energy production against the intensive environmental and ecological impacts that trigger negative consequences on traditional human populations.

Increasing energy production based on hydropower is particularly concerning and paradoxical. Once intended to mitigate climate change and adapt to its impacts, dams can become sources of significant greenhouse gas emissions and are themselves vulnerable to climate‐driven impacts (Bertassoli et al. 2021; Almeida et al. 2021). Hydropower facilities, especially run‐of‐river plants, are highly susceptible to climate‐induced changes in river flow regimes (Arias et al. 2020; Almeida et al. 2021; Caceres et al. 2021). For instance, drier conditions predicted in the eastern Amazon, driven by shifting precipitation patterns and frequent events of extreme droughts (Sorribas et al. 2016; Bottino et al. 2024), will affect power generation with sharp declines under a pessimistic scenario, up to 37% in Madeira and Tapajós (Almeida et al. 2021). The vulnerabilities of hydropower generation to climate conditions further highlight the urgency to reevaluate the projected expansion of dams in favor of alternative lower‐impact solutions.

## 5 Limitations and Perspectives

Despite offering a robust and spatially explicit framework for integrating climatic suitability and river connectivity, some uncertainties and simplifications must be acknowledged. Frugivorous fish depend strongly on forest‐river interactions and the seasonal flood pulse (Correa et al. 2016), which ties their persistence to lowland floodplain habitats. However, life‐history strategies and habitat adaptations vary widely across Amazonian fishes, and these differences can influence responses to river fragmentation and reservoir formation. For example, mountain specialists that inhabit fast‐flowing Andean or shield tributaries may be more directly affected by barriers sitting in upland regions where frugivores are largely absent. Conversely, while a few generalist or lentic tolerant species may show limited local benefits from reservoir formation, these positive effects are generally transient compared to broader negative impacts (Agostinho et al. 2008), as many Amazonian fish evolved in dynamic and free‐flowing systems. Further uncertainties can be associated with the flood‐drought dynamics that were not explicitly represented by our models. Flood amplitude, duration, or drought severity, for instance, are critical for the life‐cycle of many fish species and are expected to shift under climate change (Sorribas et al. 2016; Bottino et al. 2024). Future work should include variables that represent the alteration of natural flow regimes driven by climate change to better predict the expected distribution of species and therefore refine RCIsuit estimates and improve barrier ranking.

In addition to longitudinal fragmentation, dams impose multiple environmental changes that were not incorporated in our study and may limit our understanding of impacts on these species. Dams that form reservoirs alter flow timing and magnitude, trap sediments, modify thermal regimes, and reduce lateral connectivity with riparian forests (Agostinho et al. 2008; Franklin et al. 2024). Such alterations can reorganize community composition and disrupt ecological processes even in areas that remain structurally connected. By modeling dams solely as barriers to movement, our approach does not fully capture these additional impacts and consequently may underestimate the broader ecological costs of hydropower development, particularly of those that may appear less critical when evaluated only in terms of longitudinal connectivity. Looking forward, incorporating these additional impacts is strongly encouraged. However, this will require acknowledging clear trade‐offs, as the simultaneous consideration of multiple criteria can substantially alter dam rankings. Integrating these dimensions offers a more comprehensive evaluation of hydropower expansion while supporting decision‐makers in identifying the least detrimental options.

## 6 Conclusion

Maintaining and improving river connectivity to preserve fish dispersal and ensure long‐term population persistence is the keystone of climate adaptive strategies, prioritizing the avoidance of new barriers, while also tackling the impacts of long‐lasting structures that fragment aquatic ecosystems (Rodeles et al. 2020; Franklin et al. 2024; Fernandes et al. 2024). Freshwater conservation also implies safeguarding and monitoring adequate habitats that will be stable under climate change (Radinger et al. 2018; Herrera‐R et al. 2020; Thieme et al. 2023; Franklin et al. 2024). Our results identified locations where fish populations can persist in the absence of spatial constraints imposed by physical and climatic barriers, by integrating species distribution models to accurately identify the areas that will support fish populations while buffering impacts of climate change (Morelli et al. 2017; Wegscheider et al. 2024). By considering the potential distribution of fish simultaneously for a wide range of species, our results can also be useful to direct future efforts to quantify ecosystem services losses associated with species declines. Success, however, hinges on basin‐wide, transboundary governance, especially given that poor conservation practices in one country can undermine efforts in neighboring regions (Azevedo‐Santos et al. 2019; Cid et al. 2022). By identifying core areas that remain connected and suitable for frugivorous fish and those at risk of losing connectivity, we can prioritize rivers that are key to sustaining ecosystem functions and services. Protecting these aquatic areas generates positive cascading effects by maintaining terrestrial forests through seed dispersal by fish and by supporting human communities through the food and income these species provide. Recognizing the often‐overlooked significance of aquatic ecosystems within protected areas (Frederico et al. 2016; 2020; Bailly et al. 2021), conservation networks must be explicitly designed to include both rivers and riparian zones (Killeen and Solórzano 2008; Piczak et al. 2023) and to support local communities. Additionally, ranking the most impactful barriers for river fragmentation, our results provide innovative and applicable information for sustainable energy planning decisions that can inform policies and conservation actions that safeguard connectivity, biodiversity, and ecosystem services under changing conditions. Ultimately, where policies exist and decision‐makers are committed to maintaining or restoring freshwater systems, protection can be expanded. Our work provides a valuable tool to support these decision‐making processes by prioritizing efforts to achieve social and environmental objectives in the context of the ongoing climate crisis.

## Author Contributions

**Kátia Yasuko Yofukuji:** conceptualization, investigation, methodology, validation, visualization, writing – original draft, writing – review and editing. **Thomaz Mansini Carrenho Fabrin:** data curation, formal analysis, methodology, software, validation, visualization, writing – review and editing. **Bruno Henrique Mioto Stabile:** formal analysis, methodology, software, validation, visualization, writing – review and editing. **Angelo Antonio Agostinho:** methodology, supervision, writing – review and editing. **Céline Jézéquel:** data curation, writing – review and editing. **Valéria Flávia Batista‐Silva:** conceptualization, data curation, formal analysis, investigation, methodology, writing – review and editing. **Luiz Fernando Esser:** formal analysis, investigation, methodology, software, writing – review and editing. **José Hilário Delconte Ferreira:** data curation, investigation, methodology, writing – review and editing. **Reginaldo Ré:** methodology, software, writing – review and editing. **Pablo A. Tedesco:** funding acquisition, writing – review and editing. **João Carlos Azevedo:** conceptualization, funding acquisition, project administration, supervision, writing – original draft, writing – review and editing. **Dayani Bailly:** conceptualization, project administration, resources, supervision, writing – review and editing.

## Funding

The research was funded by the ForestFisher project: “Priority areas for conservation and restoration of Amazonian forest‐frugivorous fish interactions and associated fisheries” through the 2020–2021 Biodiversa and Water Joint Programming Initiatives (JPI) joint call for research projects, under the BiodivRestore European Research Area Network (ERA‐NET) Cofund (GA no 101003777), with the European Union and the funding organizations: Agence Nationale de la Recherche (ANR, France), Fundação para a Ciência e a Tecnologia (FCT, Portugal), Deutsche Forschungsgemeinschaft (DFG, Germany), Fundação de Amparo à Pesquisa do Estado do Amazonas (FAPEAM, Brazil), and Fundação de Apoio ao Desenvolvimento do Ensino, Ciência e Tecnologia do Estado de Mato Grosso do Sul (FUNDECT, Brazil—Process 71/001.913/2022). National funding (Portugal) was provided by FCT through a project grant of KYY. National funding (Brazil) was provided by the Fundação Araucária for the postdoctoral fellowship of TMCF and LFE, and by the Coordenação de Aperfeiçoamento de Pessoal de Nível Superior (CAPES) for the postgraduate fellowship of BHMS. This research was partially supported by national funds (Portugal) through FCT/MCTES (PIDDAC): CIMO, UIDB/00690/2020 (DOI:10.54499/UIDB/00690/2020) and SusTEC, LA/P/0007/2020 (DOI:10.54499/LA/P/0007/2020).

## Conflicts of Interest

The authors declare no conflicts of interest.

## Supporting information


**Data S1:** gcb70685‐sup‐0001‐Supinfo.pdf.

## Data Availability

The data that support the findings of this study are openly available in Zenodo at 10.5281/zenodo.18000863.
