# Supplementary material for: Anthropogenic Barriers Limit Fish Access to Essential Habitats in the Amazon in the Face of Climate Change
Source: Glob Chang Biol. 2026 Jan 2;32(1):e70685. doi: 10.1111/gcb.70685 (PMC12759175; doi:10.1111/gcb.70685)
Supplement: Supplementary file 1 — Data S1: gcb70685‐sup‐0001‐Supinfo.pdf. [file GCB-32-e70685-s001.pdf]

Supplementary material for:

Impact of anthropogenic barriers in limiting fish access to essential habitats in the Amazon in the face of climate change

Kátia Yasuko Yofukuji[1], Thomaz Mansini Carrenho Fabrin[1], Bruno Henrique Mioto Stabile[1], Angelo Antonio Agostinho[1,6], Céline Jézéquel[2], Valéria Flávia Batista-Silva[3], Luiz Fernando Esser[1], José Hilário Delconte Ferreira[4], Reginaldo Ré[4], Pablo A. Tedesco[2], João Carlos Azevedo[5]*, Dayani Bailly[1,6]*

Corresponding to: Kátia Yasuko Yofukuji (kayofukuji96@gmail.com)

*João Carlos Azevedo and Dayani Bailly should be considered joint senior author.

[1]Universidade Estadual de Maringá (UEM), Programa de Pós-graduação em Ecologia de Ambientes Aquáticos Continentais (PEA), Av. Colombo, 5790, CEP 87020-900 Maringá, PR, Brazil.
[2]Centre de recherche sur la biodiversité et l'environnement, UMR 5300, Université de Toulouse, CNRS, IRD, Toulouse, France
[3]Universidade Estadual de Mato Grosso do Sul (UEMS), Programa de Pós-graduação em Biodiversidade e Sustentabilidade Ambiental (PGBSA), Grupo de Estudo em Ciências Ambientais e Educação (GEAMBE), BR 163, 235, CEP 79982-384 Mundo Novo, MS, Brazil.
[4]Universidade Tecnológica Federal do Paraná (UTFPR), Via Rosalina Maria dos Santos, 1233, 87301-899 Campo Mourão, PR, Brazil.
[5]CIMO, LA SusTEC, Instituto Politécnico de Bragança, Campus de Santa Apolónia, 5300-253 Bragança, Portugal.
[6]Universidade Estadual de Maringá (UEM), Núcleo de Pesquisa em Limnologia, Ictiologia e Aquicultura (Nupélia), Av. Colombo, 5790, 87020-900 Maringá, PR, Brazil.

Supplementary Methods

Species selection and definition

Fish were selected based on frugivory level and with respect to their value to fisheries. Regarding the level of frugivory, a literature review was first conducted to identify species classified as having frugivorous and/or herbivorous feeding habits. Additionally, available information on the percentage of plant reproductive structures (fruits/seeds/flowers) found in the stomach contents of different species was compiled. Based on this information, the species were categorized into four levels of frugivory following the criteria proposed by Coronado-Franco et al. (2022): i) highly specialized in fruit ingestion (>75% of stomach contents composed of fruits/seeds/flowers); ii) medium-high specialization (50–75% of stomach contents); iii) medium-low specialization (25–50%); and iv) low specialization (<25%). The species were also classified according to their importance to Amazonian fisheries in the following categories: i) commercial interest; ii) commercial and subsistence interest; iii) subsistence interest; and iv) not relevant to fisheries. This classification followed the works of Cella-Ribeiro et al. (2016), Silvano (2020), Sarmiento et al. (2014), and Santos et al. (2009).

References

Cella-Ribeiro, A., Torrente-Vilara, G., Lima-Filho, J. A., & Doria, C. R. C. (2016). Ecologia e Biologia de Peixes do Rio Madeira. EDUFRO

Coronado-Franco, K.V.; Tedesco, P.A. Kolmann, M.A.; Borstein, S.R.; Evans, K.O.; Correa, S.B. (2022). Feeding habits influence species habitat associations at the landscape scale in a diverse clade of neotropical fishes. Journal of Biogeography, 49(12): 2181-2192, 2022. https://doi.org/10.1111/jbi.14490.

Santos, G.M.; Ferreira, E.J.G.; Zuanon, J. (2009). Peixes comerciais de Manaus. Manaus: INPA.

Sarmiento, J.; Bigorne, R.; Carvajal-Vallejos, F.; Maldonado, M.; Leciak, E.; Oberdorff, T. (2014). Peces de Bolivia. Bolivian fishes. Bolívia: IRD-BioFresh (EU) Plural editores, Bolivia

Silvano, R.A.M.; Hallwass, G. (2020). Uso sustentável de recursos naturais: o exemplo da pesca na Amazônia. Revista Bio Diverso, 1(1): 67-100

Table S1 List of fish species included in this study and details regarding frugivory specialization (low, mid and highly specialized), fisheries value (commercial and/or household consumption value) sample size (records mapped onto a 7 km grid covering the Amazon basin) and whether conducts longitudinal migration or not.

**Order**	**Family**	**Species**	**Sample size**	**Level of frugivory**	**Fisheries value**	**Longitudinal migration**
**Characiformes**						
	Anostomidae					
		Laemolyta proxima	220	Low	Commercial and household	no
		Leporinus fasciatus	409	Mid-low	Commercial	yes
		Leporinus friderici	698	Mid-high	Commercial and household	yes
		Megaleporinus trifasciatus	131	Highly	Commercial	yes
		Schizodon fasciatus	446	Low	Commercial and household	yes
	Bryconidae					
		Brycon amazonicus	224	Mid-high	Commercial and household	yes
		Brycon falcatus	206	Highly	Commercial and household	yes
		Brycon melanopterus	196	Mid-high	Commercial and household	yes
		Brycon pesu	261	Highly	Commercial and household	yes
	Chalceidae					
		Chalceus guaporensis	61	Low	Commercial	no
	Characidae					
		Tetragonopter	421	Low	Household	no

	us argenteus				
Erythrinidae					
	Hoplerythrinus unitaeniatus	316	Mid-high	Household	no
Hemiodontidae					
	Anodus elongatus	242	Low	Commercial and household	no
	Argonectes longiceps	88	Mid-high	Household	yes
	Hemiodus immaculatus	167	Mid-low	Household	no
	Hemiodus unimaculatus	424	Low	Household	no
Cynodontidae					
	Rhaphiodon vulpinus	366	Low	Commercial	yes
Serrasalmidae					
	Colossoma macropomum	197	Mid-high	Commercial	yes
	Myloplus asterias	136	Mid-high	Commercial and household	no
	Myloplus rubripinnis	229	Mid-high	Commercial and household	no
	Myloplus schomburgkii	120	Low	Commercial and household	no
	Myloplus torquatus	85	Mid-low	Commercial and household	no
	Mylossoma aureum	224	Highly	Commercial and household	yes
	Piaractus brachypomus	162	Highly	Commercial	yes
	Pygocentrus nattereri	430	Low	Commercial and household	yes

		Serrasalmus calmoni	43	Mid-high	Household	no
		Serrasalmus gouldingi	90	Mid-low	Household	no
		Serrasalmus manueli	103	Low	Household	no
		Serrasalmus rhombeus	756	Low	Commercial and household	no
		Serrasalmus serrulatus	59	Mid-low	Household	no
		Serrasalmus spilopleura	197	Mid-low	Household	no
		Serrasalmus striolatus	119	Highly	Household	no
	Triportheidae					
		Triportheus albus	490	Mid-low	Commercial and household	yes
		Triportheus angulatus	566	Mid-low	Commercial and household	yes
		Triportheus auritus	257	Mid-high	Commercial and household	yes
		Triportheus culter	66	Mid-low	Household	yes
		Triportheus rotundatus	70	Mid-low	Household	yes
**Cichliformes**						
	Cichlidae					
**Clupeiformes**		*Geophagus altifrons*	206	Mid-low	Household	no
		Satanoperca jurupari	560	Low	Household	no
		Uaru amphiacanthoides	73	NA	Household	no
	Pristigasteridae					
		Pellona flavipinnis	224	Low	Commercial and household	

**Osteoglossiformes**						
	Osteoglossidae					
		Osteoglossum bicirrhosum	180	Low	Commercial	no
**Siluriformes**						
	Auchenipteridae					
		Auchenipterichthys thoracatus	134	Mid-high	Household	no
	Callichthydae					
		Megalechis thoracata	221	NA	Household	no
	Doradidae					
		Oxydoras niger	193	Mid-low	Commercial and household	yes
		Pterodoras granulosus	291	Mid-low	Commercial and household	
	Pimelodidae					
		Calophysus macropterus	353	Low	Commercial and household	yes
		Leiarius marmoratus	105	NA	Commercial and household	no
		Phractocephalus hemioliopterus	142	Highly	Commercial and household	yes
		Pimelodus blochii	737	Mid-low	Commercial and household	yes
		Sorubim lima	352	NA	Commercial and household	yes
		Zungaro zungaro	142	NA	Commercial and household	yes

Barrier validation

For present-day barriers, we initially compiled 145 records from multiple datasets and publications (Table S2). After validation, using high-resolution remote sensing images and expert review, 61 records were excluded due to duplication, coordinate errors, or structures that do not block river flow, resulting in 84 barriers retained. Additionally, some barriers originally listed as “future” projects were found to have already been built. These were therefore reclassified as current. For future barriers, 330 candidate projects were compiled. These were validated against official energy expansion plans and environmental licensing inventories, and only those with documented evidence were retained, resulting in 254 barriers.

Table S2. List of barriers datasource by country

Country	Data source for anthropogenic barriers	Data source for waterfalls
Bolivia	Anderson et al. 2018; Caldas et al. 2022; RAISG, 2023; Yang et al. 2022	Lehner, 2016
Brazil	ANA, 2018; Anderson et al, 2018; Caldas et al. 2022; RAISG, 2023; Yang et al. 2022	Lehner, 2016
Colombia	Anderson et al, 2018; Caldas et al. 2022; RAISG, 2023	Lehner, 2016
Ecuador	Anderson et al, 2018; Caldas et al. 2022; RAISG, 2023; Yang et al. 2022	Lehner, 2016
Guyana		Lehner, 2016
Peu	Anderson et al, 2018; Caldas et al. 2022; RAISG, 2023; Yang et al. 2022	Lehner, 2016

References

Agência Nacional de Águas (ANA). Resolução nº 92, de 2021. Resoluções regulatórias. https://www.gov.br/ana/pt-br/legislacao/resolucoes/resolucoes-regulatorias/2021/92

Anderson, E. P., Jenkins, C. N., Heilpern, S., Maldonado-Ocampo, J. A., Carvajal-Vallejos, F. M., Encalada, A. C., Rivadeneira, J. F., Hidalgo, M., Cañas, C. M., Ortega, H., Salcedo, N., Maldonado, M., & Tedesco, P. A. (2018). Fragmentation of Andes-to-Amazon connectivity by hydropower dams. Science Advances, 4(1), eaao1642. https://doi.org/10.1126/sciadv.aao1642

Caldas, B., Thieme, M. L., Shahbol, N., Coelho, M. E., Grill, G., Van Damme, P. A., Aranha, R., Cañas, C., Fagundes, C. K., Franco-León, N., Herrera-Collazos, E. E., Jézéquel, C., Montoya, M., Mosquera-Guerra, F., Oliveira-da-Costa, M., Paschoalini, M., Petry, P., Oberdorff, T., Trujillo, F., & Tedesco, P. A. (2022). Identifying the current and future status of freshwater connectivity corridors in the Amazon Basin. Conservation

Science and Practice, 5(1). https://doi.org/10.1111/csp2.12853

Lehner, 2016. HydroFALLS v1a, Waterfall database

RAISG. Amazônia 2023: Áreas protegidas e territórios indígenas. RAISG, Brasil (2023).

Yang, X. et al. 92022) Mapping Flow-Obstructing Structures on Global Rivers. Water Resour. Res. 58, e2021WR030386.

Table S3. Sensitivity analysis of the Reach Connectivity Index (RCI) to variation in passability values. Pearson correlation quantifies concordance between alternative passability scenarios and the reference. Wilcoxon paired tests test for shifts in RCI values. Passability simulations are coded as upstXX_down_damYY_watZZ, where XX= upstream passability and YY/ZZ = downstream passability for dams and waterfalls, respectively.

Scenario	Fish group	Pearson r	Wilcoxon p-value
upst00_down_dam03_wat06	Combined	0.9764253	2.2e-16
upst00_down_dam04_wat07	Combined	0.9764675	2.2e-16
upst00_dow_dam05_wat08	Combined	0.9756720	2.2e-16
upst01_down_dam03_wat06	Combined	0.9997346	0.5114
upst01_down_dam05_wat08	Combined	0.9996253	0.08159
upst00_down_dam03_wat06	Migratory	0.9812662	2.2e-16
upst00_down_dam04_wat07	Migratory	0.9812539	2.2e-16
upst00_dow_dam05_wat08	Migratory	0.9806075	2.2e-16
upst01_down_dam03_wat06	Migratory	0.9998009	0.6003
upst01_down_dam05_wat08	Migratory	0.9997174	0.003547
upst00_down_dam03_wat06	Sedentary	0.9735161	2.2e-16
upst00_down_dam04_wat07	Sedentary	0.9735689	2.2e-16
upst00_dow_dam05_wat08	Sedentary	0.9726786	2.2e-16
upst01_down_dam03_wat06	Sedentary	0.9996855	0.3611
upst01_down_dam0	Sedentary	0.9995376	0.1713

Supplementary methods on Species distribution modelling

ODMAP Protocol

OVERVIEW

Authorship

Authors: Kátia Yasuko Yofukuji, Thomaz Mansini Carrenho Fabrin, Bruno Henrique Mioto Stabile, Angelo Antonio Agostinho, Céline Jézéquel, Valéria Flávia Batista-Silva, Luiz Fernando Esser, José Hilário Delconte Ferreira, Reginaldo Ré, Pablo A. Tedesco, João Carlos Azevedo, Dayani Bailly

Contact email: kayofukuji96@gmail.com

Model objective

We aimed to model the spatial distribution of frugivorous fish species of the Amazon basin for the current and future years under two scenarios based on two Shared Socio-economic Pathways (SSPs), moderate (SSP2-4.5) and pessimistic (SSP5-8.5).

Focal taxon

For details on each of the 52 fish species modeled, please see Table S1.

Locality

The Amazon basin integrating Bolivia, Brazil, Colombia, Ecuador, Guyana, and Peru

Scale of analysis

Spatial extent: All sub-basins from the Amazon.
Latitude: -20.2104 to 5.1396 (EPSG: 4326, WGS84)
Longitude: -79.3771 to -50.5396 (EPSG: 4326, WGS84)
Temporal extent: This study covers present-day climatic conditions and projected future scenarios for the years 2030, 2050, 2070 and 2090 consistent with the Intergovernmental Panel on Climate Change, Sixth Assessment Report (IPCC–AR6).

Boundary: Watershed boundaries of Amazon

Biodiversity data overview

Observation type: Presence records were obtained from AmazonFISH database (https://doi.org/10.6084/m9.figshare.9923762.v3), a transnational collaborative project (AmazonFish project - https://www.amazon-fish.com/) of georeferenced records from an

extensive survey of species distribution including multiple different sources (e.g. published articles, grey literature, online biodiversity databases and scientific collections from museums and universities worldwide) and field expeditions conducted during the project.

Response/data type: Presence and pseudo-absence (0s and 1s)

Type of predictors

Bioclimatic and hydrological variables (See Table S4 for full details)

Conceptual model/hypothesis

Hypothesis

We hypothesize that the distribution of Amazonian fish species is primarily determined by large-scale climatic and hydrological gradients. We expect that temperature and precipitation regimes, through their influence on water availability and seasonality, are major abiotic drivers that can shape the potential distribution of species across the basin. In addition to climate, hydrological variables related to river discharge, flow accumulation, stream order, among others, were incorporated to capture longitudinal gradients and the spatial drainage network, which are known to influence dispersal and habitat connectivity. Because the models were developed for multiple species, this approach is exploratory in nature, aiming to identify the dominant climatic-environmental correlates that define suitable conditions for fish at a basin-wide scale. Thus, we assume that variation in species occurrence among sites reflects differences in environmental suitability rather than sampling detectability. At continental scales, climatic and hydrological factors act as primary filters limiting fish distributions, whereas biotic interactions and fine-scale habitat characteristics play secondary roles.

Assumptions

Relevant biological and ecological drivers known to influence fish species distributions were included.
Occurrence records were assumed to be representative of the species' environmental tolerances across their current distribution, after filtering for spatial bias.
Species occurrence records were considered independent after duplicate removal.
Species were assumed to be at equilibrium with current environmental conditions within their accessible area.

Distributions were primarily determined by abiotic constraints and biological interactions were assumed to have a limited influence on species distributions.

SDM algorithm

Model algorithms: We used five machine learning algorithms (MLA), as follows: Maxlike (MLK), Random Forest (RF), General Linear Models (GLM), Multiple Discriminant Analysis (MDA), and Supporting Vector Machine (SVM) to predict the environmental suitability and potential distribution of species in Amazon River basin. From the outputs of such algorithms and considering the criterion adopted for evaluating the predictive performance of models, we obtained the consensus model.

Model averaging: The different SDMs provide distinct predictions of the species distribution area, generating uncertainty about which model best represents the geographic distribution of the species (Diniz-Filho et al., 2009). In order to overcome this uncertainty and minimize errors, the ensemble forecasting approach was used since it provides a consensus projection among multiple SDMs (Araújo & New, 2007).

Araújo, M., & New, M. (2007). Ensemble forecasting of species distributions. Trends in Ecology & Evolution, 22(1), 42–47. https://doi.org/10.1016/j.tree.2006.09.010

Diniz-Filho, J. A. F. Bini, L. M., Rangel, T. F., Loyola, R. D., Hof, C., Nogués-Bravo, D., & Araújo, M. B. (2009). Partitioning and mapping uncertainties in ensembles of forecasts of species turnover under climate change. Ecography, 32(6), 897–906. https://doi.org/10.1111/j.1600-0587.2009.06196.x

Model workflow

For each species, binary matrices of presence and pseudo-absence data were combined with bioclimatic and hydrological layers composed of non-collinear variables using a VIF analysis. Five machine learning algorithms were calibrated using 75% of the occurrence data for training and 25% for testing, repeated 10 times with 4-fold cross validation (40 replicates per algorithm). Model performance was assessed using the Area Under the Curve (AUC) from the Receiver Operating Characteristic (ROC) method, and only models with AUC> 0.8 were retained for ensemble forecasting. Consensus models were obtained by averaging climatic-environmental suitability across algorithms, producing a continuous suitability surface ranging from 0 to 1. Presence-absence predictions were obtained following the majority consensus rule (only cells in which at least more than half of the models indicate the presence of the species were considered occupied by the species).

Software, codes and data

Software: R version 4.4.3

Code availability: R Packages used (caretSDM and chooseGCM) are available on Luiz Esser's github.

Data availability: Data came from multiple sources with different public availability.

All fish records were obtained from the AmazonFISH public database (https://doi.org/10.6084/m9.figshare.9923762.v3). Amazon basin boundaries came from HydroBASINS, while Amazon river networks are available from HydroRIVERS.

Biodiversity data

Taxon names: Please refer to Table S1

Taxonomic reference system: Taxonomic nomenclature and species validation followed the standardized taxonomy adopted the AmazonFISH project (https://doi.org/10.1038/s41597-020-0436-4) which cross-checked all species names against FishBase and Eschmeyer's Catalog of Fishes to ensure nomenclatural accuracy. This procedure identified and corrected synonyms, typing errors, and unlisted names, and assigned a validation status for each taxon based on expert review, occurrence consistency and distributional information. Our final dataset was further verified by a specialist in Neotropical fish taxonomy to ensure the accuracy of taxonomic reliability.

Ecological level: Population-level analysis was assumed, meaning that records were treated as originating from independent populations, with variation in relative habitat suitability reflecting environmental conditions.

Biodiversity data sources: All data were obtained from AmazonFISH database (https://doi.org/10.6084/m9.figshare.9923762.v3) a transnational collaborative project, i.e. the AmazonFish project - https://www.amazon-fish.com/. of georeferenced records from an extensive survey of species distribution including multiple different sources (e.g. published articles, grey literature, online biodiversity databases and scientific collections from museums and universities worldwide) and field expeditions conducted during the project.

Sampling design: Sampling design information follows the AmazonFISH database. As the database compiles from multiple sources and collections strategies across the Amazon basin,

sampling is inherently heterogeneous, as described in the originating AmazonFISH publication (https://doi.org/10.1038/s41597-020-0436-4). Full details of data validation and source composition are presented there.

Sample size per taxon

For details on each of the 52 fish species sample size, please see Table S1.

Country/region mask: Modeling was restricted to the Amazon basin, covering portions of Bolivia, Brazil, Colombia, Ecuador, Guyana, and Peru. Basin boundary followed the HydroBASINS delineation (Lehner & Grill, 2013) which was used as a spatial mask.

Lehner, B., Grill G. (2013): Global river hydrography and network routing: baseline data and new approaches to study the world's large river systems. Hydrological Processes, 27(15): 2171–2186.

Data cleaning/filtering: Occurrence records were tested against coordinates of capitals, country centroids, equal latitude, and longitude, coordinates of research institutions, and invalid coordinates in a data cleaning process using the R package *CoordinateCleaner* (Zizka et al. 2019).

Zizka, A. et al. (2019). COORDINATECLEANER: Standardized cleaning of occurrence records from biological collection databases. Methods Ecol. Evol. 10, 744–751.

Pseudo-absence data: We generated pseudo-absences to contrast environmental information and allow the use of machine learning-based algorithms. We randomly obtained n pseudo-absences in the study area, where n is equal to the number of occurrence records of each species. This procedure was necessary to avoid imbalance issues (Japkowicz & Stephen, 2002). We built a machine learning approach to obtain occurrence probabilities using algorithms that perform their functions using pseudoabsence data, not background data (see Sillero & Barbosa, 2021). In order to build indistinguishable pseudoabsences from true absences, we selected pseudoabsences outside a surface range envelope generated with all occurrence records and selected predictor variables at these locations. This procedure guarantees that pseudoabsences are geographically and environmentally distinct from presences (see Lobo et al., 2006). To ensure that we did not fall into any imbalance issue, the number of pseudoabsences matched the number of species presence records (Japkowicz & Stephen, 2002). Thus, a binary matrix of presence (1) and pseudo-absence (0) was created for each species to feed niche models.

Japkowicz, N., & Stephen, S. (2002). The class imbalance problem: A systematic study. Intelligent Data Analysis, 6(5), 429–449. https://doi.org/10.3233/ida-2002-6504

Background data: We used presence-absence data, so no background data was generated.

Data partitioning

For each algorithm used, the species occurrence data were randomly divided into two datasets,

one comprising 75% of the data for calibration and the other with 25% for model evaluation. This process was repeated 10 times using a cross-validation system of subsampling K-folds, with K=4 (totalizing 40 repetitions). The K-fold validation divides the data into K subsets of approximately equal size and adjusts the models K times. Each time a subset is used as test data, the remaining K-1 subsets are used as training data. This procedure resulted in 40 environmental-climatic suitability projections for each algorithm and species.

Predictor variables

The distribution of the species was modeled as a function of bioclimatic and hydrological variables covering the Amazon hydrographic network. Present and future bioclimatic variables (2030, 2050, 2070, and 2090) were extracted from the WorldClim 2.1 database (http://www.worldclim.org/) with five arc minutes of spatial resolution. We also used two hydrological variables obtained from HydroSHEDS database (https://www.hydrosheds.org/products/hydrorivers). Spatial predictors are summarized in detail in Table S3.

Table S4. Information on predictors (bioclimatic and hydrological) and sources used for modelling fish species distribution for the Amazon basin.

Variable	Description	Source
**BIO1**	Annual Mean Temperature	WorldClim
**BIO2**	Mean Diurnal Range	WorldClim
**BIO3**	Isothermality (BIO2/BIO7) (×100)	WorldClim
**BIO4**	Temperature Seasonality (standard deviation ×100)	WorldClim
**BIO5**	Max Temperature of Warmest Month	WorldClim
**BIO6**	Min Temperature of Coldest Month	WorldClim
**BIO7**	Temperature Annual Range (BIO5- BIO6)	WorldClim
**BIO8**	Mean Temperature of Wettest Quarter	WorldClim
**BIO9**	Mean Temperature of Driest Quarter	WorldClim
**BIO10**	Mean Temperature of Warmest Quarter	WorldClim
**BIO11**	Mean Temperature of Coldest Quarter	WorldClim
**BIO12**	Annual Precipitation	WorldClim
**BIO13**	Precipitation of Wettest Month	WorldClim
**BIO14**	Precipitation of Driest Month	WorldClim
**BIO15**	Precipitation Seasonality (Coefficient of Variation)	WorldClim
**BIO16**	Precipitation of Wettest Quarter	WorldClim
**BIO17**	Precipitation of Driest Quarter	WorldClim
**BIO18**	Precipitation of Warmest Quarter	WorldClim
**BIO19**	Precipitation of Coldest Quarter	WorldClim
**LENGTH_KM**	length of the river reach segment	HydroSHEDS
**DIST_DN_KM**	distance from the reach outlet, i.e., the most downstream pixel of the reach, to the final downstream location along the river network, in kilometers	HydroSHEDS
**DIST_UP_KM**	distance from the reach outlet, i.e., the most downstream pixel of the reach, to the most upstream location along the river network, in kilometers	HydroSHEDS
**CATCH_SKM**	area of the catchment that contributes directly to the individual reach, in square	HydroSHEDS

	kilometers	
**UPLAND_SKM**	total upstream area, in square kilometers, calculated from the headwaters to the pour point, i.e., the most downstream pixel of the reach	HydroSHEDS
**DIS_AV_CMS**	average long-term discharge estimate for river reach, in cubic meters per second	HydroSHEDS
**ORD_STRA**	indicator of river order following the Strahler ordering system.	HydroSHEDS

Transfer data for projection

Future climate scenarios and models available in WorldClim were provided by the Intergovernmental Panel on Climate Change, Sixth Assessment Report (IPCC–AR6). Future forecasts were based on two Shared Socio-economic Pathways (SSPs), moderate (SSP2-4.5) and pessimistic (SSP5-8.5). Four atmospheric–ocean general circulation models (AOGCMs) were considered in the predictions. We used the *chooseGCM* R package (Esser et al. 2025), which helps researchers aiming to project Species Distribution Models and Ecological Niche Models to future scenarios by applying a selection routine to the General Circulation Models. The package performs a K-means cluster analysis to cluster GCMs that are similar between themselves and creates K clusters (in our analysis K = 4). Then, the package calculates which GCMs were closer to the centroid of each cluster, thus obtaining the GCMs that are the most representative of the variation within each cluster, which were: CanESM5-CanOE (Canadian Ocean Ecosystem model), GISS-E2-1-G (National Aeronautics and Space Administration - NASA, Goddard Institute for Space Studies climate model), MRI-ESM2-0 (The Meteorological Research Institute) and UKESM1-0-LL (The UK Earth System Modelling Project - UKESM).

Esser, L. F., Bailly, D., Lima, M. R., & Ré, R. (2025). *chooseGCM*: A Toolkit to Select General Circulation Models in R. Global Change Biology, 31(1). https://doi.org/10.1111/gcb.70008

Multicollinearity

The Variance Inflation Factor (VIF) was used to evaluate multicollinearity problems in our set of variables. The VIF is based on the square of the multiple correlation coefficient (R2) resulting from regressing the predictor variable against all other predictor variables. A VIF greater than 10 (as a rule of thumb) is a signal that the model has a collinearity problem (Chatterjee & Hadi 2006). We used the *vif* function in the *usdm* package to calculate the VIF value of each variable and the *vifcor* function to exclude highly collinear variables through a stepwise procedure. This approach

calculates the correlation coefficients between variables and identifies a strongly correlated pair with the highest coefficient. Then the variable with the highest VIF is excluded from the pair, and the procedure is repeated until no strongly correlated pair remains.

Chatterjee, S., & Hadi, A. S. (2006). Regression Analysis by Example. In Wiley Series in Probability and Statistics. John Wiley & Sons, Inc. https://doi.org/10.1002/0470055464

Model settings

Model settings were kept as standard using the *sdm* R package.

Model estimates

No model estimates, such as model coefficients and variable importance were obtained, since this is not in the scope of the project.

Model selection/model averaging/ensembles

Binary matrices representing the presence and pseudo-absence of each species and environmental layers corresponding to the set of non collinear variables based on VIF results for each species were used to calibrate niche models. The different SDMs provide distinct predictions of the species distribution area, generating uncertainty about which model best represents the geographic distribution of the species (Diniz-Filho et al., 2009). In order to overcome this uncertainty and minimize errors, the ensemble forecasting approach was used since it provides a consensus projection among multiple ENMs (Araújo & New, 2007). By obtaining a consensus model (CONS), errors that affect each ENM differently tend to cancel each other out, resulting in a reliable and moderate solution (Terribile et al., 2010).

Non-independence correction/analyses

We avoided spatial autocorrelation in predictors by applying the VIF routine, while occurrence data was thinned using a grid filter. In this sense, another post assessment of spatial or temporal autocorrelation was not performed, as we understand that our approach aims to reduce them at the minimum.

Threshold selection and Performance statistics

For each projection, the predictive performance of ENMs was evaluated using the Receiver Operating Characteristic (ROC) method, which generates a curve in a bivariate space by plotting the true positive rate (sensitivity) against the false positive rate (1 - true negative rate or 1 - specificity). This evaluation technique involved testing multiple candidate cut-off values to create confusion matrices, from which we selected, for each model replicate, the threshold that maximized the sum of sensitivity and specificity. The Area Under the Curve (AUC) was calculated as a measure of the predictive performance of models, independent of the decision threshold (Manel et al. 2001; Liu et al. 2005) and only models with AUC > 0.8 were combined to obtain a consensus prediction (Araújo & New, 2007). After binarization of all replicates, we combined them into an ensemble frequency map (0-1), applying a majority rule to obtain species distribution, where values higher than 0.5 indicate presences, and values lower than or equal to 0.5 represented absences.

Plausibility check

The current distribution obtained through the modeling process to each species was validated by experts.

Prediction output

Prediction unit: We interpreted the predicted response value as a climatic-environmental suitability index ranging from 0 to 1, representing the relative probability of species occurrence.

Post-processing steps

The continuous probability maps from the ensemble models were converted into binary presence-absence maps. This was achieved using a "majority consensus rule," where a grid cell was classified as "presence" only if more than half of the individual models predicted the species' presence there. The threshold for each individual model was set to maximize the sum of sensitivity and specificity. Also, a key post-processing step was the creation of an integrated index called RCIsuit. This was calculated by multiplying the normalized Reach Connectivity Index (RCI) by the mean climatic-environmental suitability score for each fish group. This composite index combines the physical accessibility of a river reach (structural connectivity) with its environmental quality (habitat suitability), providing a more ecologically complete picture of habitat availability.

Uncertainty quantification

The study addressed uncertainty in several ways. The algorithmic uncertainty was controlled by using an ensemble of five different modeling algorithms, we accounted for variability in predictions arising from the choice of model. The scenario uncertainty was addressed by projecting future scenarios using two different Shared Socio-economic Pathways (SSP2-4.5 and

SSP5-8.5) and four different General Circulation Models (GCMs) to capture a range of potential climate futures. The input data used a comprehensive, cleaned dataset from AmazonFISH and the generation of pseudo-absences outside the known environmental range of the species aimed to reduce uncertainty related to input data.

Model attributes from Species Distribution Modelling

The mean AUC value for every species modeled was 0.897 with a standard deviation of 0.026 (see Table S4 for values per species). *Chalceus guaporensis* was the species with the highest AUC (mean: 0.942; standard deviation: 0.038), while *Megalechis thoracata* was the species with the lowest AUC (mean: 0.8343; standard deviation: 0.021). *Geophagus altifrons* showed the lowest AUC variation (mean: 0.933; standard deviation: 0.013) and *Satanoperca jurupari* the highest (mean: 0.868; standard deviation: 0.058).

Table S5. Model summary information of the 52 frugivorous fish and AUC - Area Under Curve parameter used in the selection models.

Species	Mean AUC	SD AUC
Anodus elongatus	0.930450	0.027633
Argonectes longiceps	0.880500	0.035062
Auchenipterichthys thoracatus	0.878375	0.040806
Brycon amazonicus	0.908760	0.027366
Brycon falcatus	0.922815	0.024781
Brycon melanopterus	0.892470	0.044825
Brycon pesu	0.909265	0.038198
Calophysus macropterus	0.911480	0.032331
Chalceus guaporensis	0.942225	0.038348
Colossoma macropomum	0.911660	0.022983
Geophagus altifrons	0.933585	0.013774
Hemiodus immaculatus	0.905785	0.047887
Hemiodus unimaculatus	0.875800	0.051739
Hoplerythrinus unitaeniatus	0.850900	0.026799
Laemolyta proxima	0.910025	0.024388

Leiarius marmoratus	0.914750	0.015081
Leporinus fasciatus	0.864270	0.043449
Leporinus friderici	0.869013	0.040323
Megalechis thoracata	0.834380	0.021046
Megaleporinus trifasciatus	0.900430	0.031189
Myloplus asterias	0.863375	0.048555
Myloplus rubripinnis	0.855375	0.038333
Myloplus schomburgkii	0.870165	0.054369
Myloplus torquatus	0.883240	0.033243
Mylossoma aureum	0.924500	0.019983
Osteoglossum bicirrhosum	0.886080	0.041691
Oxydoras niger	0.910345	0.021664
Pellona flavipinnis	0.929995	0.015091
Phractocephalus hemioliopterus	0.912180	0.022149
Piaractus brachypomus	0.935650	0.023747
Pimelodus blochii	0.866065	0.046133
Pterodoras granulosus	0.933665	0.018925
Pygocentrus nattereri	0.908740	0.037948
Rhaphiodon vulpinus	0.907555	0.022126
Satanoperca jurupari	0.868975	0.058337
Schizodon fasciatus	0.925810	0.032051
Serrasalmus calmoni	0.911140	0.033066
Serrasalmus gouldingi	0.877800	0.036075
Serrasalmus manueli	0.920475	0.033329
Serrasalmus rhombeus	0.878565	0.040633
Serrasalmus serrulatus	0.906490	0.035655
Serrasalmus spilopleura	0.886195	0.034961

Serrasalmus striolatus	0.867235	0.024165
Sorubim lima	0.909225	0.027566
Tetragonopterus argenteus	0.889300	0.039085
Triportheus albus	0.900765	0.034060
Triportheus angulatus	0.933495	0.031696
Triportheus auritus	0.911200	0.026610
Triportheus culter	0.899705	0.026549
Triportheus rotundatus	0.846775	0.020223
Uaru amphiacanthoides	0.940080	0.021142
Zungaro zungaro	0.888550	0.021912

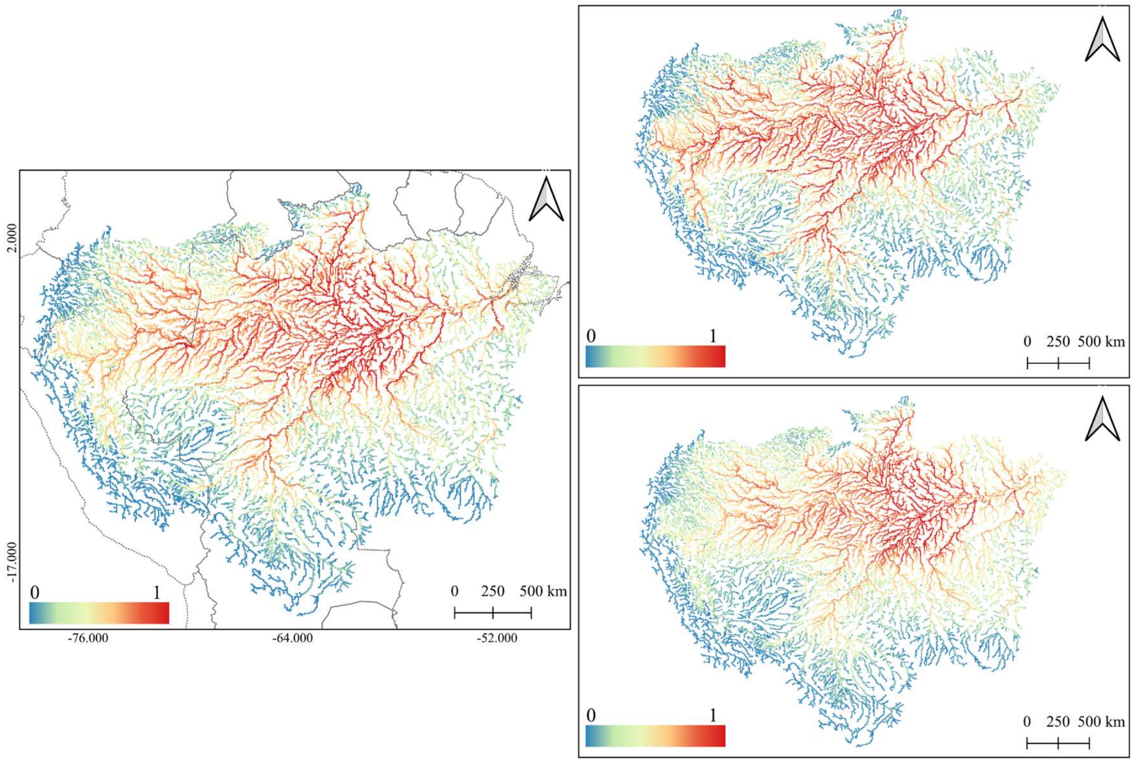

Fig S1. Maps of climatic-environmental suitability derived from the consensus models for the current time. The maps display habitat suitability for the combined fish group (left), migratory species (top right), and sedentary species (bottom right). Suitability values range from 0 to 1, where 1 represents ideal environmental conditions and 0 indicates suboptimal conditions.

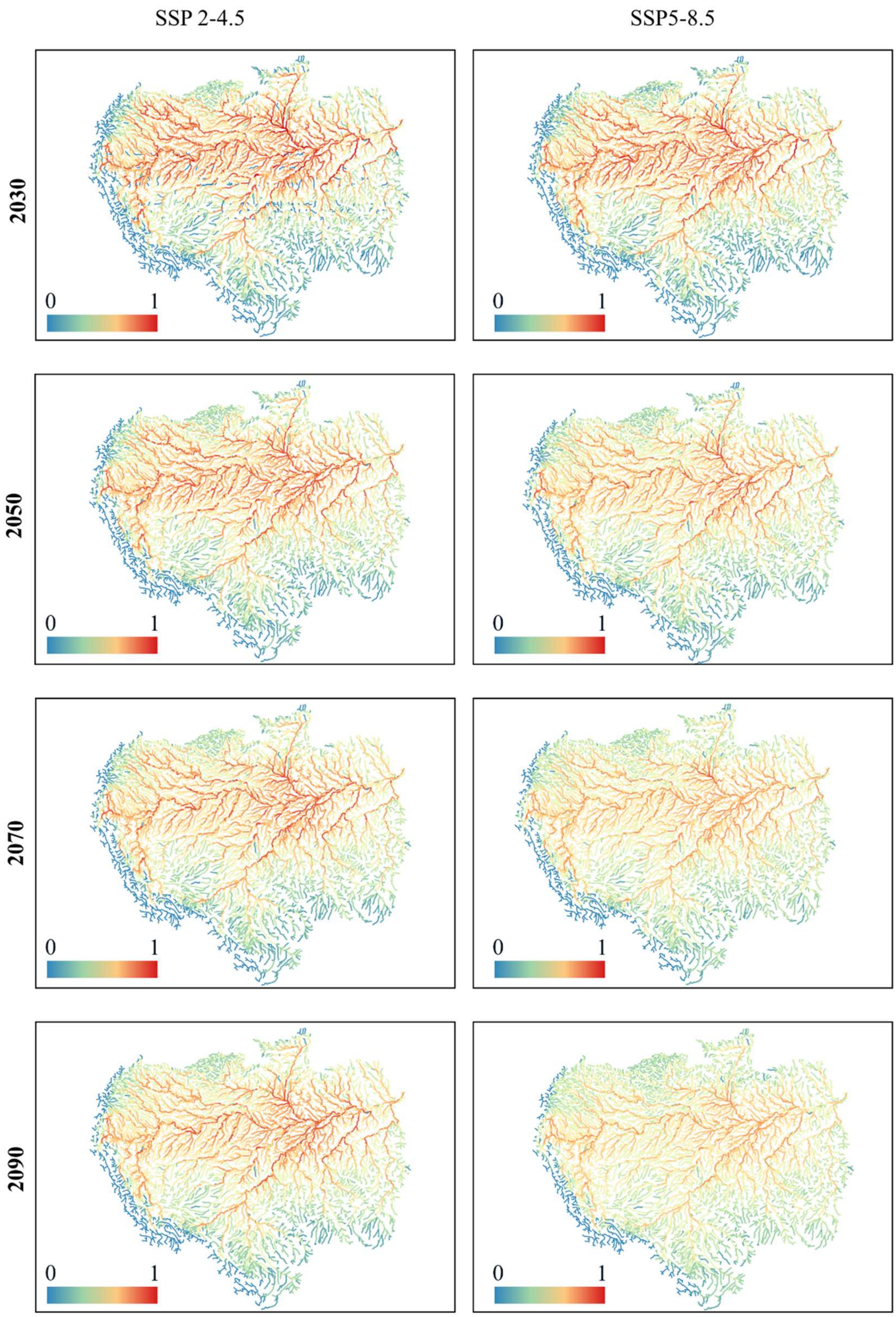

Fig. S2 Maps of climatic-environmental suitability derived from the consensus models for migratory fish species for future times (2030-2100), under two Shared Socioeconomic Pathways (SSP2-4.5 represents a moderate scenario and SPP5-8.5 represents a pessimistic scenario). Suitability values range from 0 to 1, where 1 represents ideal environmental conditions and 0 indicates suboptimal conditions.

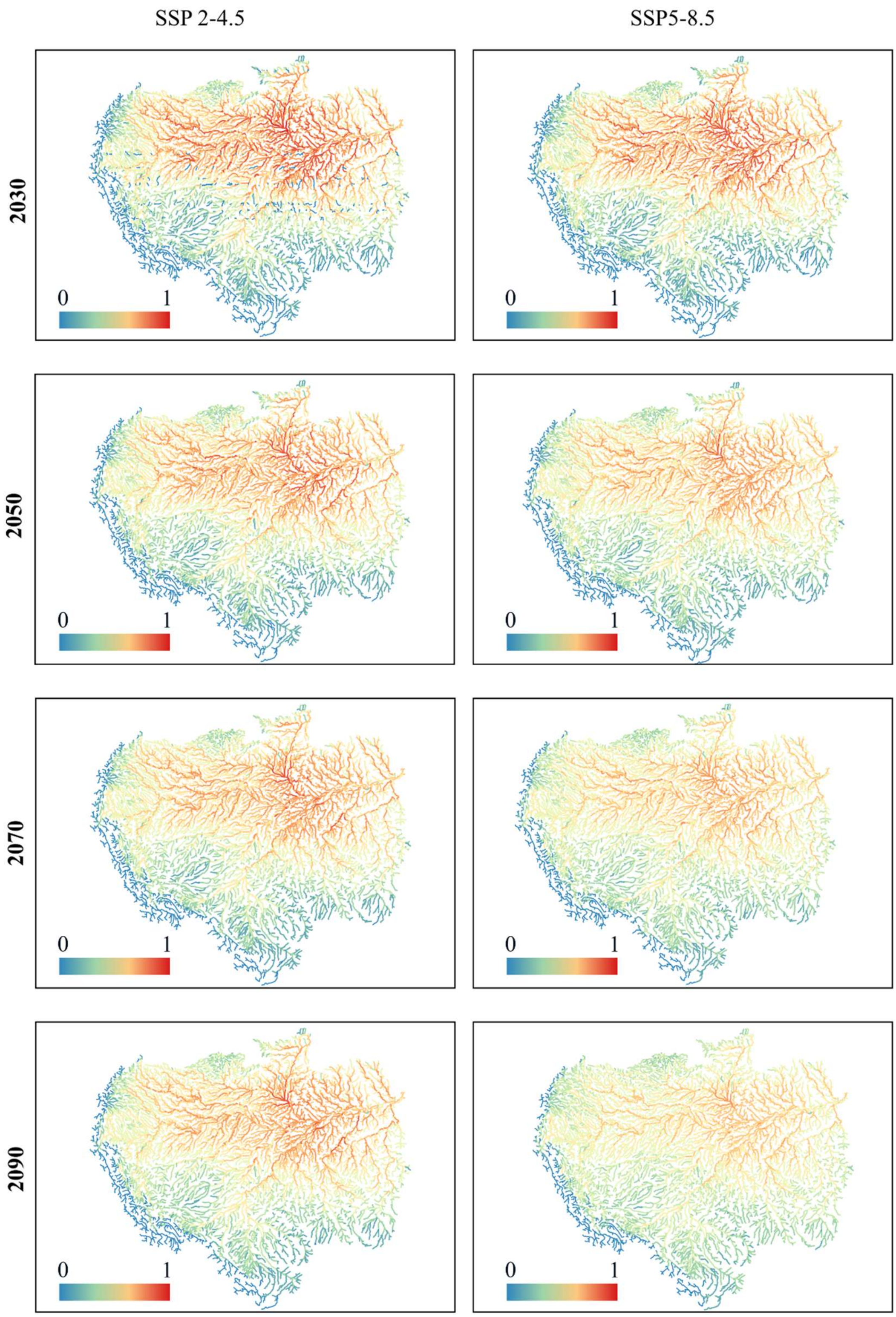

Fig. S3 Maps of climatic-environmental suitability derived from the consensus models for sedentary fish species for future times (2030-2100), under two Shared Socioeconomic Pathways (SSP2-4.5 represents a moderate scenario and SPP5-8.5 represents a pessimistic scenario). Suitability values range from 0 to 1, where 1 represents ideal environmental conditions and 0 indicates suboptimal conditions.

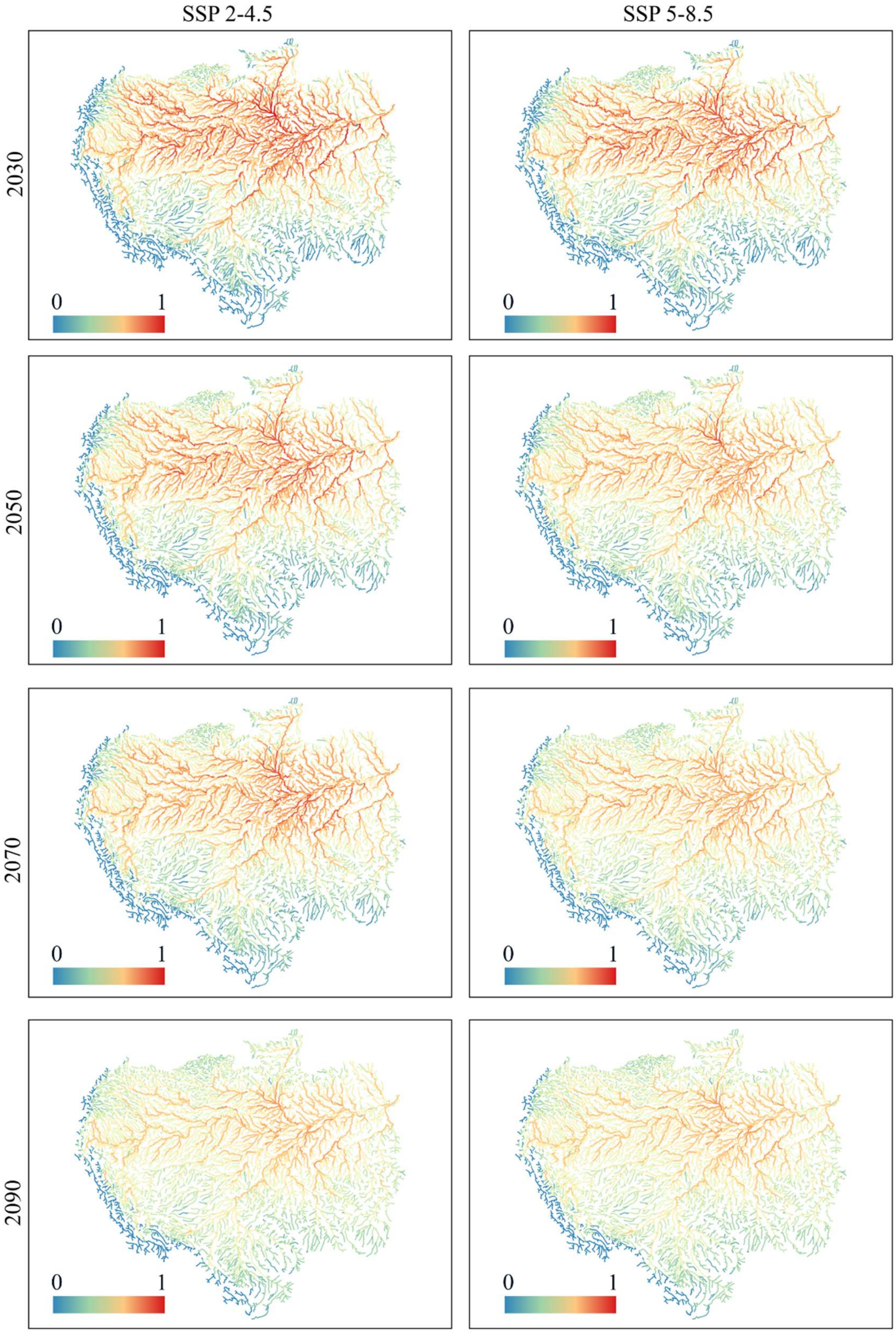

Fig. S4 Maps of climatic-environmental suitability derived from the consensus models for combined fish group for future times (2030-2100), under two Shared Socioeconomic Pathways (SSP2-4.5 represents a moderate scenario and SPP5-8.5 represents a pessimistic scenario). Suitability values range from 0 to 1, where 1 represents ideal environmental conditions and 0 indicates suboptimal conditions.

Table S6 Summary statistics of RCIsuit values by fish group for the baseline and under moderate and pessimistic climate change scenarios for "current barrier" and "future barrier" setups.

Group	Scenario	Year	Mean		Median		Min		Max	
Combined	**baseline**		0.2272614		0.2540948		2.509177e-05		0.6573463	
Migratory	**baseline**		0.2325299		0.254196		1.990519e-07		0.6661124	
Sedentary	**baseline**		0.2182991		0.2281614		3.064132e-05		0.6497447	
						**Scenario**				
			**moderate**				**pessimistic**			
			**Mean**	**Median**	**Min**	**Max**	**Mean**	**Median**	**Min**	**Max**
Combined	**current**	2024	0.220867	0.135515	1.80E-7	0.631808	0.220867	0.135515	1.8098E-7	0.631808
		2030	0.222027	0.219620	1.58E-7	0.597006	0.216108	0.214190	1.5834E-7	0.580160
		2050	0.214959	0.236019	1.508E-7	0.564548	0.204302	0.236589	1.5080E-7	0.534670
		2070	0.211949	0.243497	1.5080E-7	0.555118	0.200042	0.248318	1.3572E-7	0.513180
		2090	0.211695	0.249529	1.3572E-7	0.540431	0.191747	0.242006	2.1866E-7	0.480915
Migratory	**current**	2024	0.225782	0.080355	8.8328E-9	0.640234	0.225782	0.080355	8.8328E-9	0.640234
		2030	0.220310	0.178664	8.8328E-9	0.605601	0.222979	0.220295	3.1366E-8	0.591294
		2050	0.221796	0.244891	2.9154E-8	0.575327	0.210555	0.244550	1.4521E-8	0.541924
		2070	0.218298	0.252289	2.9154E-8	0.566565	0.206387	0.252556	1.4521E-8	0.522369
		2090	0.218325	0.258990	3.1366E-8	0.552050	0.197538	0.244239	1.0165E-7	0.490612
Sedentary	**current**	2024	0.205599	0.123675	3.7640Ee-7	0.624502	0.205599	0.123675	3.7640E-7	0.624502
		2030	0.213396	0.181920	3.2935E-7	0.587723	0.208687	0.187882	3.1366E-7	0.576050
		2050	0.207575	0.207267	3.1366E-7	0.553917	0.197548	0.210282	3.1366E-7	0.527540
		2070	0.205	0.21494	3.136	0.54	0.193	0.22429	2.195	0.50

			091	7	6E-7	5863	190	3	6E-7	3255
		2090	0.204534	0.219612	2.8230E-7	0.532888	0.185492	0.228162	1.8820E-7	0.477215
Combined	**future**	2030	0.172117	0.039737	4.0643E-8	0.517416	0.167517	0.038320	4.5159E-8	0.502816
		2050	0.165404	0.038466	4.0643E-8	0.489286	0.156143	0.037029	5.4191E-8	0.463391
		2070	0.162201	0.038291	5.1933E-8	0.481113	0.151618	0.036715	5.4309E-8	0.444765
		2090	0.161800	0.038847	3.8385E-8	0.468383	0.144423	0.035511	7.8447E-8	0.416802
Migratory	**future**	2030	0.170840	0.035660	1.2036E-9	0.524865	0.172217	0.040978	2.5997E-9	0.512466
		2050	0.169940	0.040908	1.2035E-9	0.498627	0.160109	0.039814	5.5392E-9	0.469678
		2070	0.166263	0.040989	3.8739E-9	0.491033	0.155543	0.039503	1.0778E-8	0.452730
		2090	0.166043	0.041354	7.5389E-9	0.478454	0.147892	0.037586	6.5857E-8	0.425207
Sedentary	**future**	2030	0.166603	0.036729	5.1662E-8	0.509371	0.162442	0.036034	5.6359E-8	0.499254
		2050	0.160504	0.037024	5.1870E-8	0.480071	0.151859	0.035660	5.1662E-8	0.457211
		2070	0.157814	0.036880	5.6193E-8	0.473091	0.147378	0.034890	7.5145E-8	0.436164
		2090	0.157217	0.037075	4.2269E-8	0.461846	0.140676	0.033758	9.2045E-8	0.413595

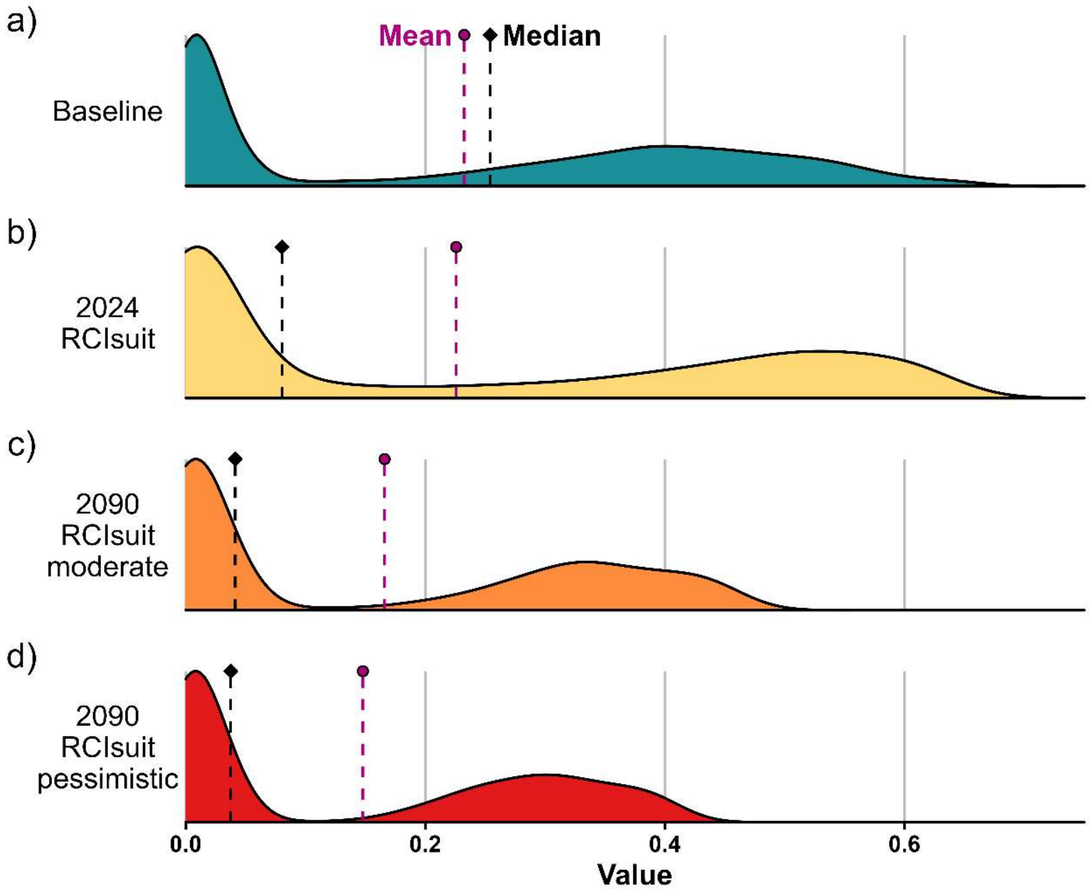

Figure S5 Distribution of RCIsuit values for the migratory fish group under four simulations: baseline (represents natural fragmentation only by waterfalls combined with current climatic-environmental suitability), 2024 RCIsuit (corresponds to the combination of existing dams and waterfalls under current climatic-environmental suitability), and 2090 - moderate and pessimistic (represents the addition of proposed barriers to the current set under changing climatic-environmental suitability of the end of the century). RCIsuit values integrate the accessibility of habitats and their climatic-environmental suitability, ensuring that regions favorable for fish dispersal were identified based on both criteria. Each density plot represents the distribution of values with summary statistics: dashed black line marks the median, and the dashed purple line indicates the mean value for each distribution.

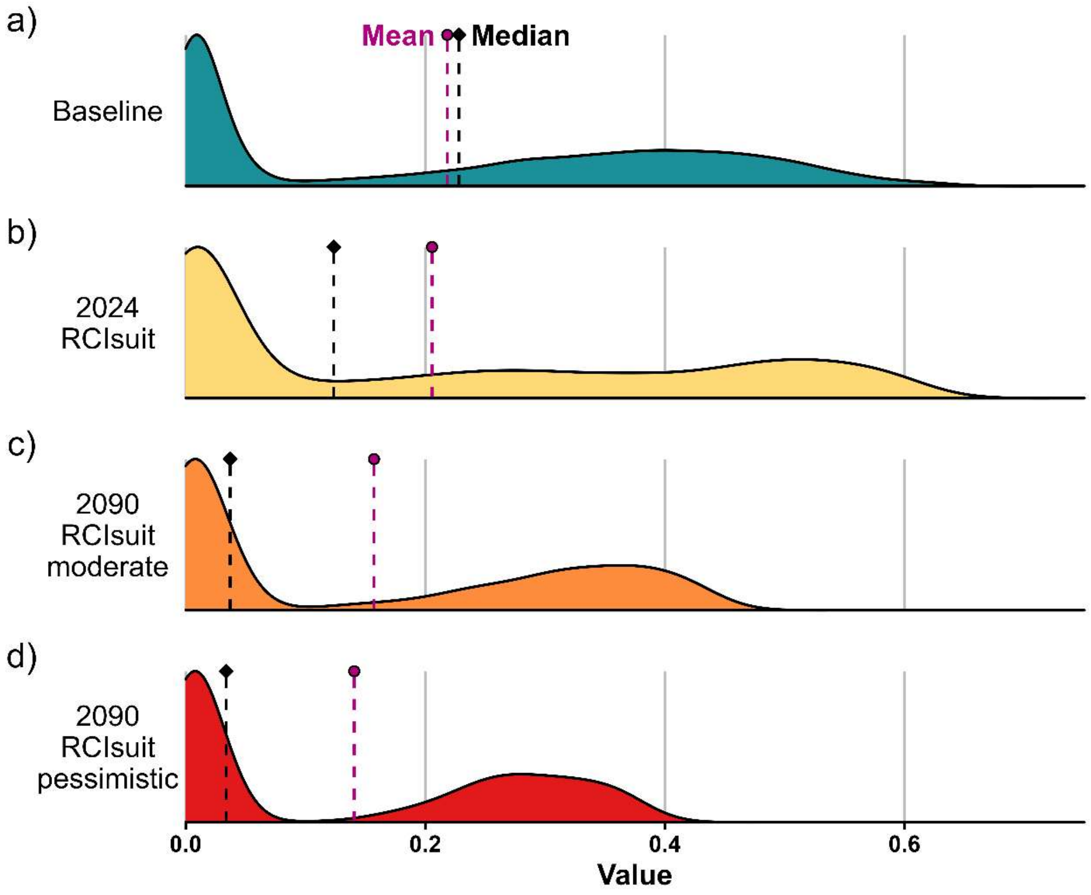

Figure S6 Distribution of RCIsuit values for the sedentary fish group under four simulations: baseline (represents natural fragmentation only by waterfalls combined with current climatic-environmental suitability), 2024 RCIsuit (corresponds to the combination of existing dams and waterfalls under current climatic-environmental suitability), and 2090 - moderate and pessimistic (represents the addition of proposed barriers to the current set under changing climatic-environmental suitability of the end of the century). RCIsuit values integrate the accessibility of habitats and their climatic-environmental suitability, ensuring that regions favorable for fish dispersal were identified based on both criteria. Each density plot represents the distribution of values with summary statistics: dashed black line marks the median, and the dashed purple line indicates the mean value for each distribution.

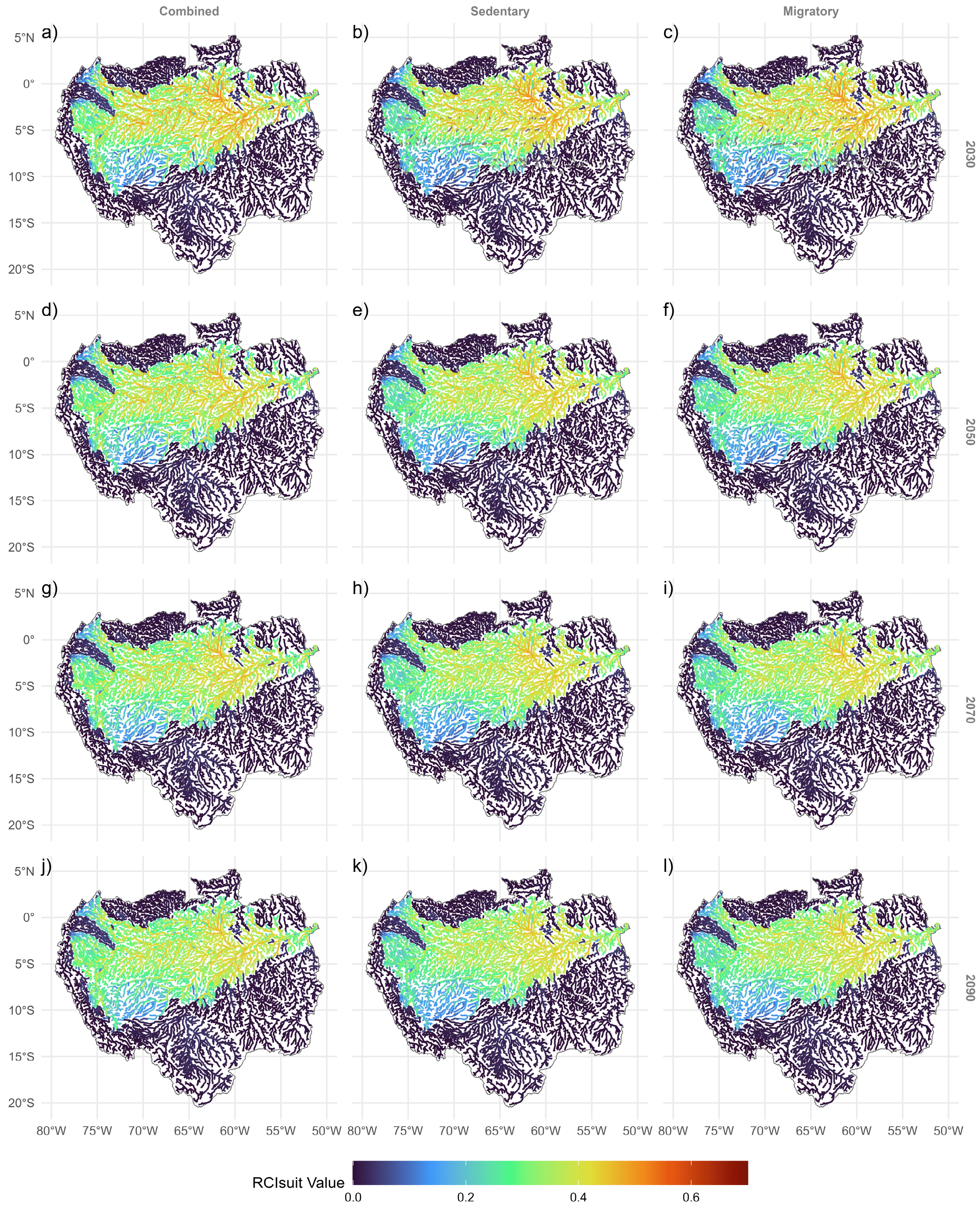

Figure S7 Spatial distribution of the RCIsuit index across the Amazon Basin for combined, migratory, and sedentary fish groups under the moderate climate change scenario incorporating future barrier scenarios. The color gradient represents river connectivity and climatic-environmental habitat suitability, ranging from red (high connectivity and high habitat suitability) to dark blue (low connectivity and low habitat suitability). The future barrier set includes both operational barriers and those planned for construction within projected energy matrix expansion.

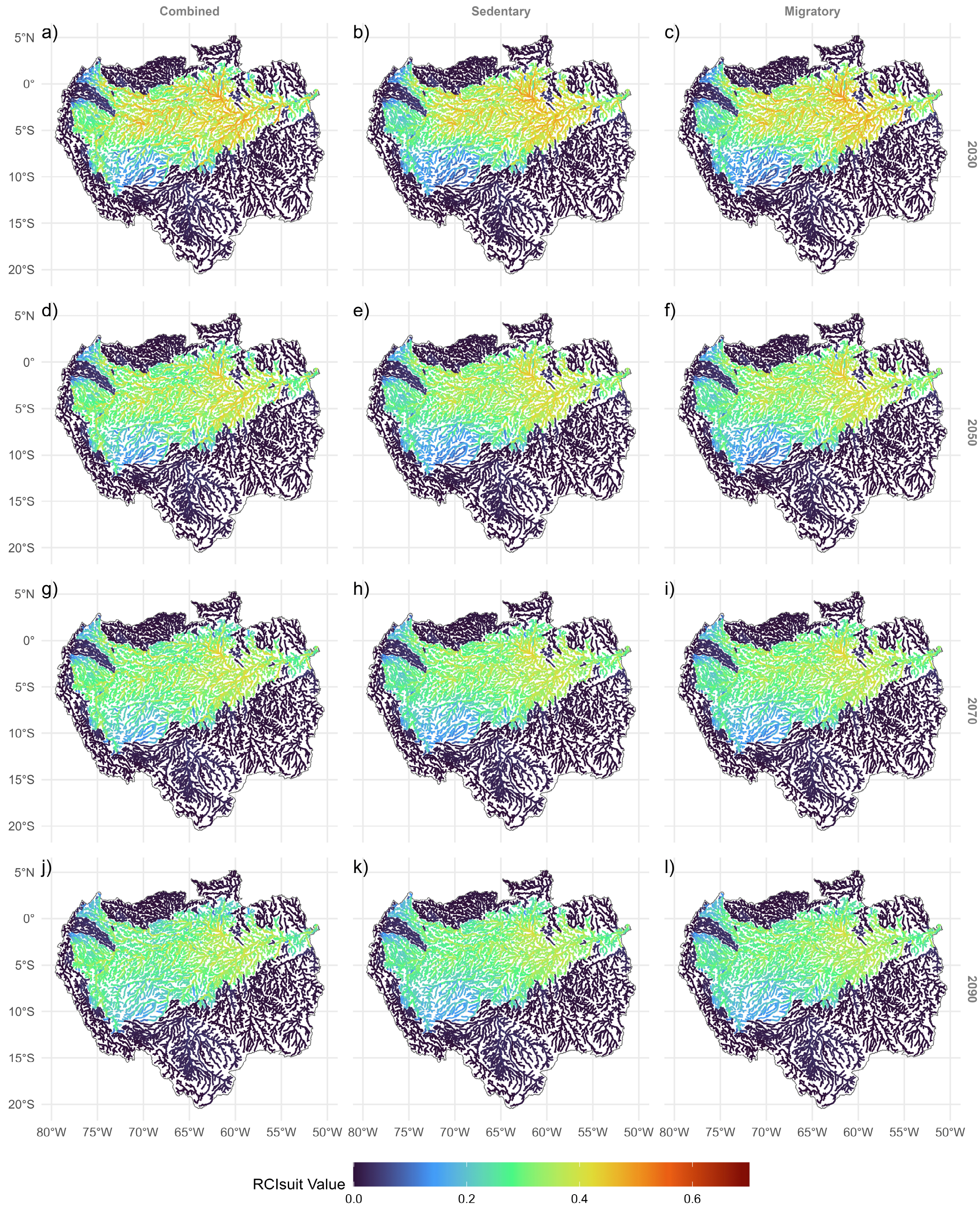

Figure S8. Spatial distribution of the RCIsuit index across the Amazon Basin for combined, migratory, and sedentary fish groups under the pessimistic climate change scenario incorporating future barrier scenarios. The color gradient represents river connectivity and climatic-environmental habitat suitability, ranging from red (high connectivity and high habitat suitability) to dark blue (low connectivity and low habitat suitability). The future barrier set includes both operational barriers and those planned for construction within projected energy matrix expansion.
